# Pain, sensory acceptability, and treatment compliance following cryotherapy and topical anesthetics in children: A randomized clinical trial

**DOI:** 10.1371/journal.pone.0362074

**Published:** 2026-11-03

**Authors:** Emine Zehra Aydın Çetin, Bilal Özmen

**Affiliations:** Ondokuz Mayıs University, Faculty of Dentistry, Department of Pediatric Dentistry, Samsun, Türkiye; Indiana University School of Dentistry, UNITED STATES OF AMERICA

## Abstract

This randomized clinical trial aimed to compare the effects of cryotherapy, anesthetic spray, and gel on pain, anxiety, physiological responses, taste acceptability, and treatment compliance in children aged 7–10 years undergoing their first dental treatment. 100 healthy children were randomly allocated into four groups: 10% lidocaine spray, 20% benzocaine gel, cryotherapy with distilled water cooled to +4 °C, and lidocaine spray cooled to +4 °C. Following topical application, buccal infiltration anesthesia was administered. Anxiety, pain, and physiological responses were assessed using the Modified Dental Anxiety Scale and Facial Image Scale, Wong-Baker FACES Pain Rating Scale, and pulse-oximeter. Taste acceptability and treatment compliance were evaluated using a three-point Hedonic Scale and the FRANKL Scale. Repeated Measures ANOVA, Mann-Whitney U, and Kruskal-Wallis tests were applied. No significant differences were observed among the groups in anxiety, pain, pulse rate, oxygen saturation, or taste acceptability (p > 0.05). Pulse rate increased from the pre-operative to the post-operative period in all groups (p = 0.006), with no significant group × time interaction. FRANKL scores were significantly higher in the cryotherapy-distilled water group compared with the gel and cooled-spray groups (p = 0.001), a finding considered exploratory. Pain and anxiety outcomes following cryotherapy did not differ significantly from those of the topical anesthetic agents, while treatment compliance was higher, suggesting that cryotherapy may be a promising non-pharmacological option, pending confirmation in non-inferiority trials.

**Trial registration:**

ClinicalTrials.gov NCT07198022

## Introduction

During dental treatments, the most stressful and anxiety-inducing factor, and the most common reason for missing or postponing appointments, is the fear of pain and injury from injections. This condition, frequently encountered in childhood, tends to persist into later life. One in four adults has an injection phobia, and one in 20 patients avoids dental treatment due to this phobia [1]. In pediatric patients, intense resistance to injections, hysterical reactions, and anxiety attacks may hinder the delivery of planned dental care, ultimately leading to untreated caries and a consequent decline in oral health-related quality of life [1–3]. To minimize discomfort and improve patient cooperation, topical anesthetics are routinely applied before needle insertion, aiming to reduce pain perception at the injection site and provide both psychological and physical relief [4]. The rationale for this approach is grounded in the neurophysiological mechanisms underlying dental pain transmission.

Tooth innervation is mediated by branches of the trigeminal nerve (the fifth cranial nerve), specifically the maxillary nerve (n. maxillaris) in the maxilla and the mandibular nerve (n. mandibularis) in the mandible [5]. Nociceptive stimuli originating from dental tissues activate peripheral nociceptors, triggering the generation and propagation of action potentials along sensory neurons. This process is primarily governed by voltage-gated sodium channels, which play a central role in neuronal excitability and pain signaling [5,6].

Local anesthetics achieve their analgesic effect by reversibly blocking voltage-gated sodium channels, thereby preventing sodium influx and inhibiting both the initiation and propagation of action potentials. By interrupting nociceptive signal transmission at the peripheral nerve level, these agents effectively reduce pain perception during dental procedures [7,8]. However, the injection itself may cause discomfort, as commercial local anesthetic solutions, particularly those containing epinephrine, are formulated at an acidic pH to prolong shelf life, which is considered one of the causes of the burning sensation experienced during injection [9]. In this context, topical anesthetics are widely recommended prior to injection, particularly in children and anxious patients, as a supportive strategy to attenuate superficial nociceptive input and enhance comfort during local anesthetic administration [4].

Pain and anxiety are multidimensional experiences influenced by both psychological and physiological factors [10,11]. While self-reported scales provide valuable insight into subjective pain perception and emotional responses, physiological parameters, such as pulse rate and oxygen saturation, serve as objective markers of autonomic nervous system activation during dental procedures [11–13].

Recently, cryotherapy has gained increasing popularity as a non-pharmacological technique for alleviating post-operative pain [14]. In dentistry, cryotherapy has been used as an adjunctive pain management strategy following tooth extractions, periodontal surgical procedures, and implant surgeries [15]. Beyond post-operative applications, cryotherapy has also been explored as an alternative topical anesthetic. A recent systematic review and meta-analysis by Saeedi et al. synthesized the available randomized evidence on cryotherapy and topical anesthetics for intraoral injection pain, reporting that these approaches may reduce injection-related discomfort [16]. Moreover, cryotherapy has also been reported to improve pulpal anesthesia success rates in adolescent patients undergoing endodontic treatment, supporting its broader analgesic potential in dental practice [17]. Nevertheless, the available evidence has largely focused on pain outcomes, leaving the broader sensory and behavioral dimensions of these interventions, such as taste acceptability and treatment compliance, insufficiently addressed in the literature.

In dental settings, pediatric patients commonly exhibit a natural avoidance of materials that induce unpleasant sensory experiences, such as pain, burning sensations, or unfavorable taste. These sensory perceptions may significantly influence children's acceptance of dental procedures and their willingness to cooperate during treatment [18]. Children's behavioral responses and treatment compliance are equally important clinical outcomes alongside pain reduction, yet the impact of topical anesthetic agents and cryotherapy on these dimensions remains insufficiently investigated. Despite the widespread use of these agents in pediatric dentistry, no study to date has systematically evaluated taste acceptability and treatment compliance alongside pain outcomes in a comparative four-arm design. Therefore, this study aimed to evaluate the effects of cryotherapy compared with routinely used topical anesthetic agents on injection-related pain, taste acceptability, and treatment compliance in pediatric patients, alongside dental anxiety and physiological responses, to provide a more comprehensive assessment of the pediatric patient experience extending beyond pain measurement to incorporate sensory and behavioral dimensions.

## Materials and Methods

### Study design and ethical approval

This randomized clinical trial was conducted at the Department of Pediatric Dentistry, Ondokuz Mayıs University. The study protocol was approved by the Ondokuz Mayıs University Clinical Research Ethics Committee (Approval No: 2024/316) and the Turkish Medicines and Medical Devices Agency (No: E-24931227-514.04.02-5719207). The trial was registered at ClinicalTrials.gov (NCT07198022) and conducted in accordance with the principles of the Declaration of Helsinki. Participants were recruited between 25/09/2025 and 15/01/2026. Written informed consent was obtained from both children and their legal guardians before enrollment.

### Sample size calculation

Sample size was calculated using G*Power (version 3.1.9.7) with the “ANOVA: Fixed effects, omnibus, one-way” procedure for four independent groups. An effect size of Cohen's f = 0.50 was estimated from the means and standard deviations of pain scores reported by Dhingra et al. [19]. Assuming α = 0.05 and a power of 95%, the minimum required total sample size was 76 participants (19 per group). To compensate for potential dropouts, 100 participants (25 per group) were enrolled.

### Participants and eligibility criteria

Children aged 7–10 years who were literate, classified as ASA physical status I-II, exhibited positive behavior (FRANKL scores 3–4), and had a Modified Dental Anxiety Scale (MDAS) score < 19 were eligible for inclusion. Additional inclusion criteria were an indication for treatment of primary teeth, no known allergy to the active ingredients of the topical anesthetic agents, and no prior dental treatment experience.

Children who otherwise met the inclusion criteria were excluded if the tooth to be treated was symptomatic (spontaneous or acute pain, swelling, or a sinus tract). Accordingly, all included children were asymptomatic at the time of treatment, and the indicated treatments included restorative treatment, pulpotomy, or extraction of primary maxillary molars.

### Randomization and blinding

Eligible participants were randomly allocated to four parallel groups (n = 25 per group) using sequentially numbered opaque sealed envelopes prepared by an independent researcher to ensure allocation concealment. Participants were not informed of the existence of the alternative interventions and had no prior dental treatment experience, so they encountered the assigned agent without any basis for comparison and were unaware of their group allocation. Due to the distinct physical characteristics of the topical materials, operator blinding was not feasible. Therefore, the study was designed as a single-blinded randomized clinical trial, in which participants were blinded to group allocation. Outcome assessments were performed by an independent evaluator blinded to the intervention groups, constituting an assessor-blinded design.

### Interventions

Participants were allocated to one of four intervention groups:

- Group 1: Application of 10% lidocaine hydrochloride topical spray (Locanest®, Avixa İlaç San. ve Tic. Ltd. Şti., Turkey).
- Group 2: Application of 20% benzocaine topical gel with strawberry flavor (Vision®, Anadolu Diş Deposu San. ve Tic. A.Ş., Turkey).
- Group 3: Application of cryotherapy using distilled water stored at +4 °C and applied immediately after removal from refrigeration.
- Group 4: Application of cooled topical anesthetic spray (10% lidocaine hydrochloride, Locanest®) stored at +4 °C prior to application.

All topical agents were applied to the dried mucosal surface using a cotton pellet, gently pressed onto the injection site for 20 seconds, and followed by verbal confirmation of perceived numbness by the child prior to injection.

After the patients confirmed adequate topical anesthesia, buccal infiltration anesthesia in the maxillary molar region was administered by a single operator using 4% articaine hydrochloride with 1:100,000 epinephrine (Ultraver D-S Forte, Haver, Turkey). A 2-mL disposable syringe (Ayset, Turkey) with a 27-i, 1½-inch (38-mm) needle was used, and 1.5 mL of anesthetic solution was deposited slowly, approximately 60 seconds. For standardization, physiological measurements recorded during injection were obtained at the moment of initial needle penetration, before anesthetic solution deposition.

### Outcome measures

- The primary outcome was pain perception during local anesthetic injection, assessed using the Wong-Baker FACES Pain Rating Scale (WBPRS)

Secondary outcomes included:

- Dental anxiety, assessed using the Modified Dental Anxiety Scale (MDAS) and Facial Image Scale (FIS)
- Treatment compliance, assessed using the FRANKL Behavior Rating Scale during local anesthetic injection.
- Taste acceptability, assessed using the Three-Point Hedonic Scale
- Changes in physiological parameters (pulse rate and oxygen saturation), measured using a pulse oximeter (G-Life YK-80B, Xuzhou Yongkang Electronic Science Technology Co., Ltd., China) at three time points: before topical application (pre-operative), during topical application (peri-operative), and during local anesthetic injection (post-operative).

### CONSORT flow of participants

No participants were excluded after randomization. All randomized participants received the allocated intervention, completed the study protocol, and were included in the final analysis. Additionally, no adverse events were observed in any participant during or immediately after the procedures. The flow of participants through the study phases was reported in accordance with the CONSORT guidelines [20].

### Statistical analysis

All statistical analyses were performed using IBM SPSS Statistics version 21.0 (IBM Corp., Armonk, NY, USA). The distribution of continuous variables was assessed using the Shapiro-Wilk test. Pulse rate and oxygen saturation, which met the assumption of normality, were analysed using a two-way repeated-measures analysis of variance (ANOVA), with measurement period (baseline, topical application, injection) as the within-subject factor and group as the between-subject factor; the group × time interaction was included to test whether changes over time differed among the interventions. Sphericity was assessed using Mauchly's test, and the Greenhouse-Geisser correction was applied when the assumption was violated. Partial eta-squared (ηp²) was reported as the effect size. When a significant main effect of time was found, pairwise comparisons between measurement periods were performed with Bonferroni adjustment. Between-group differences were further examined using Tukey's HSD test and are presented as mean differences with 95% confidence intervals.Ordinal and non-normally distributed outcomes (MDAS, FIS, WBPRS, Three-Point Hedonic Scale, and FRANKL scores) were compared among the four groups using the Kruskal-Wallis test. Effect sizes were calculated as epsilon-squared (ε² = H/(n − 1)) for all Kruskal-Wallis analyses. Where a significant difference was found, pairwise comparisons were performed with Bonferroni adjustment. The Mann-Whitney U test was used to compare anxiety scores (MDAS and FIS) between girls and boys. Baseline age was compared across groups using one-way ANOVA, and sex distribution using the Pearson chi-square test. As the WBPRS is a 0–10 scale with equal intervals, pairwise mean differences with Tukey-adjusted 95% confidence intervals were additionally calculated to quantify the magnitude and precision of between-group differences in the primary outcome.

Continuous variables are presented as mean ± standard deviation and ordinal variables as median (first-third quartile). Because medians of short ordinal scales may be identical across groups despite differences in score distributions, means with 95% confidence intervals are provided as supplementary descriptive statistics; all inferences were based on nonparametric tests. Secondary outcomes were considered exploratory, and no adjustment was made for multiple testing across outcomes. Exact p-values are reported, and statistical significance was set at p < 0.05. The de-identified individual participant data and data dictionary are provided as Supporting Information (S1 Dataset).

## Results

The flow of participants through the study phases is presented in Fig 1. A total of 100 individuals were included in the study (48 females and 52 males). Gender distribution was comparable across groups, with no statistically significant differences observed (Pearson chi-square, χ²(3) = 3.205, p = 0.361). Age profiles were also similar, with a mean age of approximately 8 years and no significant differences among groups (One-way ANOVA, F(3,96) = 0.822, p = 0.485). Baseline demographic characteristics by study group are summarized in Table 1.

**Table 1:** Age and sex distribution of participants across the study groups.

Groups	Female, n (%)	Male, n (%)	Total	Age (Mean ± SD)	95% CI (Lower Bound- Upper Bound)
**Group 1**	9 (36%)	16 (64%)	25	8.36 **±** 1.036	7.93-8.78
**Group 2**	15 (60%)	10 (40%)	25	8.32 **±** 1.07	7.88-8.76
**Group 3**	13 (52%)	12 (48%)	25	8.52 **±** 1.0	8.10-8.93
**Group 4**	11 (44%)	14 (56%)	25	8.04 **±** 1.27	7.51-8.56
**Total**	**48** (48%)	**52** (52%)	**100**	**8.31 ± 1.1**	**8.09-8.52**

**SD:** Standard Deviation. **CI:** 95% confidence interval

**Fig 1:**
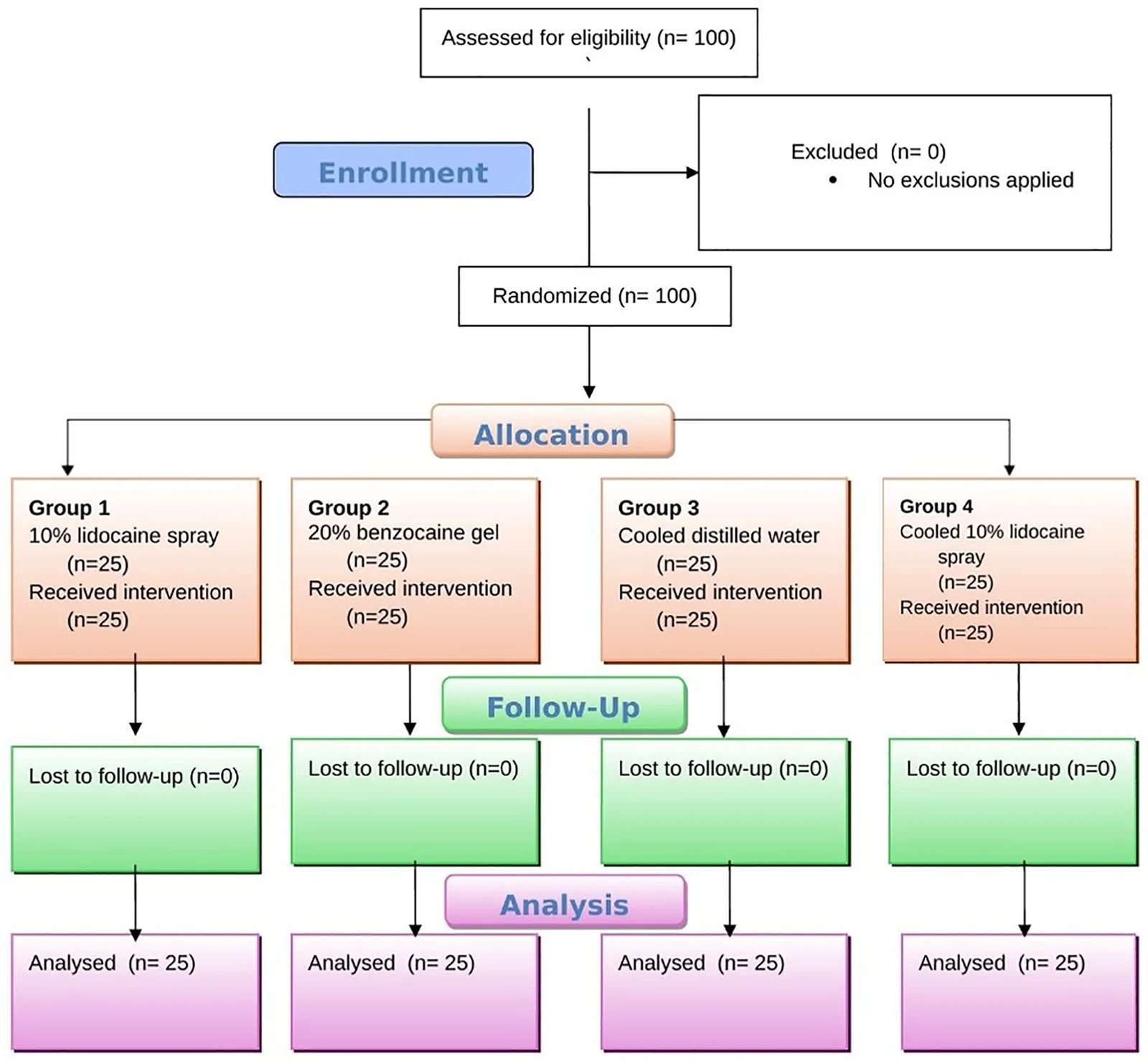
CONSORT flow diagram of participant recruitment.

Pulse rate values obtained during the pre-operative, peri-operative, and post-operative periods are presented in Table 2. As Mauchly's test indicated a violation of sphericity (χ²(2) = 8.10, p = 0.017), Greenhouse-Geisser corrected results are reported. A significant main effect of time was observed (F(1.85, 177.50) = 5.56, p = 0.006, ηp² = 0.055). Bonferroni-adjusted pairwise comparisons showed that pulse rate increased significantly from baseline to injection (mean difference 4.45 beats/min, 95% CI 0.78–8.12; p = 0.012), whereas the differences between baseline and topical application (p = 0.185) and between topical application and injection (p = 0.287) were not significant. Neither the main effect of group (F(3, 96) = 1.23, p = 0.303, ηp² = 0.037) nor the group × time interaction (F(5.55, 177.50) = 1.10, p = 0.361, ηp² = 0.033) was significant, indicating that changes in pulse rate over time did not differ among the interventions. Between-group mean differences with 95% confidence intervals are presented in S1 Table.

**Table 2:** Pulse rate (beats/min) according to study groups and measurement periods.

*Groups*	*n*	*Pre-operative*	*Peri-operative*	*Post-operative*
*Group 1*	25	88.08 ± 12.70	91.88 ± 14.03	93.16 ± 17.95
*Group 2*	25	94.28 ± 15.44	95.08 ± 15.66	102.04 ± 18.52
*Group 3*	25	94.84 ± 11.06	98.24 ± 12.96	96.04 ± 8.80
*Group 4*	25	93.52 ± 15.40	95.36 ± 13.81	97.28 ± 14.82
*Total*	100	92.68 ± 13.84	95.14 ± 14.12	97.13 ± 15.61^a^

Values are presented as mean ± standard deviation. Repeated-measures ANOVA results are reported in the text.

^a^Significantly higher than the pre-operative value (Bonferroni-adjusted p = 0.012).

Oxygen saturation values are presented in Table 3. As the assumption of sphericity was violated (Mauchly's test, χ²(2) = 12.90, p = 0.002), Greenhouse-Geisser corrected results are reported. No significant main effect of time (F(1.78, 170.37) = 2.96, p = 0.061, ηp² = 0.030), main effect of group (F(3, 96) = 1.84, p = 0.144, ηp² = 0.054), or group × time interaction (F(5.32, 170.37) = 1.46, p = 0.202, ηp² = 0.044) was observed. Between-group mean differences with 95% confidence intervals are presented in S1 Table.

**Table 3:** Oxygen saturation (%) according to study groups and measurement periods.

*Groups*	*n*	*Pre-operative*	*Peri-operative*	*Post-operative*
*Group 1*	25	98.36 ± 0.76	98.32 ± 0.80	98.56 ± 0.65
*Group 2*	25	98.16 ± 0.62	97.92 ± 0.70	98.28 ± 0.61
*Group 3*	25	98.24 ± 0.72	97.60 ± 2.08	98.28 ± 0.68
*Group 4*	25	98.12 ± 1.05	98.44 ± 0.77	98.36 ± 0.95
*Total*	100	98.22 ± 0.80	98.07 ± 1.26	98.37 ± 0.73

Values are presented as mean ± standard deviation.

Patient-reported outcome measures and behavioral assessment scores across the study groups are summarized in Table 4. MDAS scores ranged from 5 to 14 across all groups. Intergroup comparisons did not reveal any statistically significant differences in anxiety levels as assessed by MDAS (H(3) = 1.725, p = 0.631, ε² = 0.017). Similarly, no statistically significant differences were observed among the groups in FIS scores (H(3) = 3.410, p = 0.333, ε² = 0.034). No significant differences in anxiety scores were observed between girls and boys (MDAS: U = 1124.0, Z = −0.86, p = 0.388; FIS: U = 1076.5, Z = −1.26, p = 0.207). Consistent with these findings, pain scores evaluated using the WBPRS scale showed no statistically significant differences between the groups (H(3) = 3.609, p = 0.307, ε² = 0.036). All pairwise 95% confidence intervals for differences in WBPRS scores included zero, with the largest difference observed between the benzocaine gel and cryotherapy groups (1.28 points, 95% CI −0.99 to 3.55) (S2 Table).

**Table 4:** Anxiety, pain, taste acceptability, and behavior scores among the study groups.

Scale	Statistic	Group 1	Group 2	Group 3	Group 4	p
MDAS	Median (Q1-Q3)	10 (7.5-12)	9 (8.5-11)	10 (8-12)	10 (9-12.5)	0.631
	Mean (95% CI)	9.68(8.60-10.76)	9.48(8.84- 10.12)	9.80 (8.82-10.78)	10.24(9.28-11.20)	
FIS	Median (Q1-Q3)	1 (1-3)	2 (1-3)	1 (1-3)	1 (1-3)	0.333
	Mean (95% CI)	1.84 (1.42-2.26)	2.28 (1.83-2.73)	1.84 (1.42-2.26)	1.92 (1.42-2.42)	
WBPRS	Median (Q1-Q3)	2 (0-5)	2 (0-6)	0 (0-4)	2 (2-4)	0.307
	Mean (95% CI)	3.12 (1.86-4.38)	3.60 (2.15-5.05)	2.32 (0.96-3.68)	2.96 (2.03-3.89)	
Three-Point Hedonic Scale	Median (Q1-Q3)	2 (1-3)	2 (1.5-3)	3 (2-3)	2 (1-3)	0.142
	Mean (95% CI)	2.04 (1.69-2.39)	2.16 (1.83-2.49)	2.48 (2.21-2.75)	2.00 (1.66-2.34)	
FRANKL	Median (Q1-Q3)	3 (3-4)	3 (3−3)	4 (3–4)^a^	3 (3-3.5)	**0.001**
	Mean (95% CI)	3.36 (3.16-3.56)	3.20 (3.03-3.37)	3.72 (3.53-3.91)	3.24 (3.06-3.42)	

p values from the Kruskal-Wallis test. Q1-Q3, first and third quartiles; CI, confidence interval; MDAS, Modified Dental Anxiety Scale; FIS, Facial Image Scale; WBPRS, Wong-Baker FACES Pain Rating Scale.

Medians (Q1-Q3) are the primary summary measures, means (95% CI) are supplementary.

^a^Significantly higher than the benzocaine gel (adjusted p = 0.001) and cooled lidocaine spray (adjusted p = 0.003) groups (Bonferroni-adjusted pairwise comparisons).

Taste acceptability assessed using the Three-Point Hedonic Scale did not differ significantly among the groups (H(3) = 5.444, p = 0.142, ε² = 0.055).

Behavioral assessments using the FRANKL Behavior Rating Scale indicated positive to definitely positive behavior across all groups. A statistically significant difference was observed among the groups in terms of FRANKL scores (Kruskal-Wallis, H(3) = 17.649, p = 0.001; ε² = 0.178). Bonferroni-adjusted pairwise post hoc comparisons revealed significant differences between the gel and cryotherapy (distilled water) groups (adjusted p = 0.001) and between the cooled-spray and cryotherapy (distilled water) groups (adjusted p = 0.003). the comparison with the topical spray group did not reach statistical significance (adjusted p = 0.054)

## Discussion

Dental anxiety is frequently associated with needle-related pain, and the use of topical anesthetic agents has therefore been recommended to minimize discomfort during local anesthetic administration [4]. In pediatric dental practice, various topical anesthetics are routinely applied for this purpose, among which 10% lidocaine hydrochloride spray and 20% benzocaine are among the most commonly used agents [4,21]. In recent years, growing interest in non-pharmacological approaches, alongside conventional pharmacological methods for controlling injection pain, has led to increased investigation into the effectiveness of cryotherapy and cooled topical anesthetic formulations [22].

The present randomized clinical trial evaluated the effects of cryotherapy and commonly used topical anesthetic agents on pediatric patients undergoing buccal infiltration anesthesia, extending beyond pain measurement to incorporate sensory acceptability and behavioral compliance alongside injection-related pain. Dental anxiety and physiological responses were assessed as secondary outcomes.

While the analgesic efficacy of cryotherapy and cooled topical anesthetics has been recently synthesized in the literature [16], the broader patient experience, including sensory acceptability and behavioral compliance, has remained largely unaddressed. The present study was therefore designed to extend beyond pain measurement by incorporating these dimensions as key secondary outcomes.

In the present study, both the Modified Dental Anxiety Scale (MDAS) and the Facial Image Scale (FIS) were used to evaluate dental anxiety in children before topical anesthetic application. Questionnaire-based assessments are widely accepted psychometric tools due to their practicality and ease of use in clinical settings, and the MDAS is among the most commonly used instruments for assessing dental anxiety. The MDAS is a brief, easily understandable five-item scale rated on a five-point Likert scale [23]. In addition, visual projective methods are particularly advantageous in younger pediatric populations, as they allow children to express their immediate emotional state without relying on advanced verbal or cognitive skills. The Facial Image Scale, consisting of five facial expressions ranging from very happy to very unhappy, is valid from age 3 [24]. The combined use of these scales enabled the establishment of a standardized pre-procedural baseline, ensuring that children’s anxiety levels were comparable and not influenced by situational factors. This approach minimized the potential confounding effect of pre-existing anxiety and allowed a more accurate evaluation of the impact of the topical anesthetic intervention on children’s responses. Similarly, the baseline symptomatic status of the tooth may influence pain perception and pre-procedural anxiety, as pulpal inflammation lowers the stimulation threshold of nociceptors and may result in hyperalgesia [25], and pain associated with irreversible pulpitis has been linked to higher levels of dental anxiety [26]. In the present study, all included teeth were clinically asymptomatic, and pain and behavioral compliance were assessed during local anesthetic injection, before any dental treatment was initiated, thereby minimizing the potential confounding effect of differing clinical diagnoses and treatment types.

Besides situational factors, dental anxiety in children is influenced by several individual and experiential determinants, including previous dental experiences. Boka et al. reported that children with prior dental treatment experiences, particularly those accompanied by pain, tended to demonstrate increased anxiety, heightened pain perception, and reduced cooperation during subsequent local anesthetic administration [27].

Based on this evidence, only children with no previous dental treatment experience were included in the present study to eliminate the potential confounding effects of past positive or negative dental experiences on anxiety, pain perception, and behavioral responses. This approach was adopted to achieve a more homogeneous baseline and to allow a more precise evaluation of the effects of the topical anesthetic interventions.

In physiological measurements, unlike subjective methods, anxiety levels are quantified using specialized devices that measure values such as pulse, blood pressure, and oxygen saturation (SpO_2_). The somatic effects of anxiety manifest through sympathetic activation of the autonomic nervous system. This activation has a multisystemic impact on the gastrointestinal, cardiovascular, respiratory, and endocrine systems [13]. Among physiological measurement methods, pulse rate is among the most frequently used parameters for evaluating anxiety and pain in dental applications [12,13]. The literature has shown that increased anxiety and stress levels during dental treatment are associated with simultaneous increases in pulse rate and blood pressure [13,28].

Physiological measurements allow for comparable results regarding the effects of anxiety across different time intervals, such as before, during, and after treatment. In this study, physiological measurements of children were taken at three time points: before the procedure, during topical anesthesia application, and during injection, using pulse and SpO_2_ values. Pulse rate increased significantly from the pre-operative to the post-operative period, which is consistent with local anesthetic injection being one of the most anxiety-provoking stage of dental treatment [1]. However, the absence of a significant group × time interaction indicates that this increase did not differ among the interventions. Although the mean pulse rate in the cryotherapy group was numerically lower at the time of injection than during topical application, this pattern was not statistically supported and should not be interpreted as a differential physiological response.

Regarding oxygen saturation, SpO_2_ values showed similar patterns across all study groups at each measurement time, suggesting that the evaluated interventions did not result in clinically meaningful changes in oxygen saturation. Rafatjou et al. reported statistically significant differences in SpO_2_ levels across interventions; however, these changes were of limited magnitude and did not consistently reflect procedural anxiety, suggesting that oxygen saturation may be less sensitive to short-term anxiety-related physiological changes during pediatric dental procedures [29]. This indicates that, although minor and transient, reductions in SpO_2_ may occur during stressful clinical procedures; oxygen saturation alone does not reliably reflect patients’ subjective anxiety levels, and physiological oxygenation remains stable.

Chilakamuri et al. evaluated injection-related pain in children aged 7–9 years by comparing 5% lignocaine gel with cryotherapy (ice application) using the Colored Analogue Scale and reported that cryotherapy was more effective at reducing injection pain [30]. Maganur et al. assessed the efficacy of topical anesthetic gel, cryotherapy (ice), and cooled topical anesthetic gel before injection in children aged 6–12 years, using the Wong-Baker Scale (WBPRS) and Face, Legs, Activity, Cry, Consolability (FLACC) scales, and demonstrated that cooling of the anesthetic agent resulted in a significant reduction in pain perception [31]. In contrast, Havale et al., who assessed children aged 6–10 years, reported no consistent superiority of cryotherapy over topical anesthetic agents across various materials [32].

In the present study, pain perception was evaluated using the Wong-Baker FACES Pain Rating Scale following the application of cooled distilled water, benzocaine gel, lidocaine spray, and cooled lidocaine spray; however, no significant differences were observed among the study groups. Consistent with our findings, Lakshmanan et al. conducted a randomized clinical trial in children aged 7–10 years comparing the effectiveness of benzocaine gel and cryotherapy (ice application) before intraoral injection. The authors reported no significant difference in pain perception between the groups, as assessed by WBPRS scores [33]. Similarly, Anantharaj et al. (2020) evaluated the efficacy of cryotherapy (ice) and benzocaine in children aged 9–10 years using both WBPRS and Sound-Eye-Motor (SEM) methods. Likewise, they found no significant difference between the two approaches [34]. Additionally, Ninawe and Anija evaluated the pre-injection efficacy of benzocaine gel and cryotherapy (ice application) in children aged 5–10 years using the WBPRS. They reported no significant difference between the two interventions [35]. To this end, in subjective pain assessment tools such as the WBPRS, factors including children’s tendency to use a limited range of scores, individual differences in pain perception, and the relatively short duration of pain experienced during injection may make it difficult to detect statistically significant differences between groups. In this context, the absence of a statistically significant difference in WBPRS scores between the gel and spray applications in the present study was consistent with findings reported in the existing literature. Given that cooled distilled water yielded pain and anxiety outcomes that did not differ significantly from those of the pharmacological agents, its low cost, ready availability, and absence of pharmacological side effects may make it a practical option in pediatric dental practice.

Beyond pain control, treatment compliance represents a critical outcome in pediatric dentistry, as uncooperative behavior may compromise the quality and continuity of dental care [1]. In the present study, the cryotherapy-distilled water group demonstrated significantly higher FRANKL scores compared with the gel and cooled spray groups, indicating better treatment cooperation. This finding may be clinically relevant, as it suggests that applying cooled distilled water may positively influence children's behavioral responses during injection, independent of its analgesic effect. The sensory novelty of cold temperature stimulation, combined with the absence of pharmacological taste, may contribute to a more tolerable procedural experience and improved cooperation during treatment. However, given the large number of secondary outcomes evaluated and the absence of multiplicity adjustment, this finding should be interpreted as exploratory.

To assess taste perceptions, a Three-Point Hedonic Scale adapted from Chen et al. (1996) was used [36]. This approach allows for an age-appropriate evaluation of children's sensory experience without requiring excessive cognitive effort. This study represents one of the first attempts to systematically evaluate the taste acceptability of cryotherapy alongside conventional topical anesthetic agents. Our results indicated that taste preferences were largely neutral across all groups, suggesting that none of the interventions resulted in undesirable sensory responses. These findings may have important clinical implications; the sensory acceptability of cryotherapy did not differ significantly from that of the conventional pharmacological agents, suggesting that it was well accepted by children. In children who are sensitive or averse to the taste of pharmacological preparations, cooled distilled water may therefore offer a viable non-pharmacological option without compromising sensory tolerance. While taste acceptability should be interpreted alongside other factors such as anxiety and pain perception, its systematic assessment contributes to a more comprehensive evaluation of the pediatric patient experience.

Maganur et al. have investigated the use of topical anesthetic gels cooled before intraoral injections and found that cooling these agents may enhance their analgesic effectiveness [31].. However, to the best of our knowledge, no studies have specifically evaluated the effect of cooling topical anesthetic sprays on injection-related pain perception. In the present study, no significant differences were observed between the cooled and non-cooled spray formulations for pain control or taste perception, suggesting that cooling may not confer additional clinical benefit when topical anesthetics are administered in spray form. This finding may be attributed to differences in formulation characteristics, including reduced mucosal contact time and lower tissue retention compared with gel-based preparations.

Vasakova et al. reported in their studies of preschool children that girls were more affected by anxiety [37]. Similarly, Vlad et al. noted that girls were more prone to anxiety in their study conducted on children aged 6–9 years [38]. In contrast, Karaca and Sirinoğlu Capan, in their study involving children aged 5–8 years, found that dental anxiety was independent of gender [39]. In the present study, no significant relationship was found between the gender of the children and their anxiety levels. These differing results in the literature are thought to stem from variations in sample characteristics, age groups, anxiety assessment methods used, and inclusion criteria.

In the current study, children aged seven years and older were included to ensure sufficient cognitive ability for reliable self-reporting of pain, anxiety, and sensory perceptions. This age threshold has been widely adopted in pediatric dental research, as children above this age are generally able to comprehend assessment scales and provide consistent responses.

Only patients requiring maxillary buccal infiltration anesthesia were selected to standardize the injection procedure and minimize procedural variability. Since topical anesthetics exert their effect primarily within the superficial mucosal layers, with a penetration depth of approximately 2–3 mm, buccal infiltration anesthesia involves a substantially shorter needle penetration depth than mandibular block techniques, whereas inferior alveolar nerve block (IANB) typically requires deep needle penetration (around 20–25 mm) [40]. Therefore, buccal infiltration may entail less extensive soft-tissue penetration and potentially lower injection-related discomfort [41,42].

Several limitations should be acknowledged. Regarding the study design, the sample size calculation was based on a large assumed effect size (Cohen's f = 0.50). Therefore, although the enrolled sample exceeded the calculated requirement and no participants were lost to follow-up, the study may have been underpowered to detect smaller but clinically meaningful between-group differences, particularly in pain perception. Accordingly, the confidence intervals for WBPRS differences were wide, and differences of up to approximately 3.5 points could not be excluded. Thus, the non-significant findings, particularly for pain, may reflect insufficient power rather than a true absence of differences and should be interpreted with caution. In addition, the trial was not designed to establish equivalence or non-inferiority and the absence of significant differences should not be interpreted as evidence of equivalence among the interventions. Multiple secondary outcomes were evaluated without adjustment for multiplicity, which increases the risk of type I error; thus, the significant difference in treatment compliance should be regarded as exploratory and requires confirmation in future trials. The parallel-group design may have allowed individual differences in pain thresholds to obscure between-group differences, although such trials more closely resemble clinical conditions [9]. Furthermore, the sealed-envelope randomization method may introduce a potential risk of selection bias; future studies should consider centralized allocation concealment.

Regarding the intervention, the short topical application time of 20 seconds may have been insufficient to achieve optimal mucosal anesthesia, which may have contributed to the lack of significant differences among the groups. The study was also limited to a specific age group and a single injection technique, which may restrict generalizability.Regarding the participants and outcome measures, only children with positive baseline behavior (FRANKL scores 3–4) were included. As a result, treatment compliance could vary only between positive and definitely positive behavior, which may have produced a ceiling effect and limited the ability to detect differences; moreover, the findings may not be generalizable to children with negative baseline behavior. Finally, pain and taste assessments relied on subjective measures, which, although validated, may be influenced by individual perception and developmental factors.

## Conclusions

Within the limitations of this trial, cryotherapy with cooled distilled water produced injection-related pain, dental anxiety, physiological, and taste acceptability outcomes that did not differ significantly from those of 10% lidocaine spray, 20% benzocaine gel, and cooled 10% lidocaine spray in children aged 7–10 years undergoing their first dental treatment. In an exploratory analysis, treatment compliance, assessed with the FRANKL scale, was significantly better in the cryotherapy group than in the gel and cooled-spray groups. Cooling the lidocaine did not result in significant differences compared with the room-temperature formulation for either pain control or taste acceptability. Cryotherapy with cooled distilled water may therefore represent a simple, inexpensive, and well-tolerated non-pharmacological approach in pediatric dentistry, with a possible advantage in behavioural cooperation; as the trial was not designed to test equivalence or non-inferiority, this potential should be confirmed in adequately powered non-inferiority trials. These conclusions are drawn from a single-centre trial restricted to one age range, one injection technique, and children with positive baseline behaviour, and require confirmation in larger multicentre trials powered to detect smaller between-group differences.

## Supporting information

S1 Dataset De-identified individual participant data. (SAV)

S2 Dataset De-identified individual participant data and data dictionary. (XLSX)

S1 Table Pairwise between-group comparisons of pulse rate and oxygen saturation. Mean differences with 95% confidence intervals (Tukey's HSD), averaged across measurement periods. (DOCX)

S2 Table Pairwise differences in WBPRS pain scores between study groups. Mean differences with Tukey-adjusted 95% confidence intervals.
(DOCX)

S1 Checklist CONSORT 2025 checklist of information. (DOCX)

S1 File Study protocol Study protocol approved by the Ondokuz Mayıs University Clinical Research Ethics Committee (English translation). (DOCX)
