# Supplementary material for: Pain, sensory acceptability, and treatment compliance following cryotherapy and topical anesthetics in children: A randomized clinical trial
Source: PLoS One. 2026 Nov 3;21(11):e0362074. doi: 10.1371/journal.pone.0362074 (PMC13730363; doi:10.1371/journal.pone.0362074)
Supplement: Supplementary file 1 [file pone.0362074.s003.docx]

**S1 Table. Pairwise between-group comparisons of pulse rate and oxygen saturation (averaged across measurement periods; Tukey's HSD)**

| Comparison | Pulse rate: mean difference (95% CI) | p | SpO₂: mean difference (95% CI) | p |
| --- | --- | --- | --- | --- |
| Lidocaine spray vs. benzocaine gel | -6.09 (-15.17 to 2.98) | 0.301 | 0.29 (-0.17 to 0.76) | 0.355 |
| Lidocaine spray vs. cryotherapy | -5.33 (-14.41 to 3.74) | 0.420 | 0.37 (-0.09 to 0.84) | 0.160 |
| Lidocaine spray vs. cooled lidocaine spray | -4.35 (-13.42 to 4.73) | 0.596 | 0.11 (-0.36 to 0.57) | 0.932 |
| Benzocaine gel vs. cryotherapy | 0.76 (-8.32 to 9.84) | 0.996 | 0.08 (-0.38 to 0.54) | 0.969 |
| Benzocaine gel vs. cooled lidocaine spray | 1.75 (-7.33 to 10.82) | 0.958 | -0.19 (-0.65 to 0.28) | 0.720 |
| Cryotherapy vs. cooled lidocaine spray | 0.99 (-8.09 to 10.06) | 0.992 | -0.27 (-0.73 to 0.20) | 0.441 |
