# Supplementary material for: Pain, sensory acceptability, and treatment compliance following cryotherapy and topical anesthetics in children: A randomized clinical trial
Source: PLoS One. 2026 Nov 3;21(11):e0362074. doi: 10.1371/journal.pone.0362074 (PMC13730363; doi:10.1371/journal.pone.0362074)
Supplement: Supplementary file 2 [file pone.0362074.s004.docx]

**S2 Table. Pairwise differences in WBPRS pain scores between study groups. Mean differences with Tukey-adjusted 95% confidence intervals.**

| Comparison | Mean difference | 95% CI |
| --- | --- | --- |
| Lidocaine spray vs. Benzocaine gel | −0.48 | −2.75 to 1.79 |
| Lidocaine spray vs. Cryotherapy | 0.80 | −1.47 to 3.07 |
| Lidocaine spray vs. Cooled spray | 0.16 | −2.11 to 2.43 |
| Benzocaine gel vs. Cryotherapy | 1.28 | −0.99 to 3.55 |
| Benzocaine gel vs. Cooled spray | 0.64 | −1.63 to 2.91 |
| Cryotherapy vs. Cooled spray | −0.64 | −2.91 to 1.63 |
