# Supplementary material for: Pain, sensory acceptability, and treatment compliance following cryotherapy and topical anesthetics in children: A randomized clinical trial
Source: PLoS One. 2026 Nov 3;21(11):e0362074. doi: 10.1371/journal.pone.0362074 (PMC13730363; doi:10.1371/journal.pone.0362074)
Supplement: Supplementary file 4 [file pone.0362074.s006.docx]

**Project Summary**

Dental injections are among the most common sources of fear and anxiety in children and may negatively influence treatment compliance and future dental attendance. Topical anesthetic agents are routinely used to reduce injection discomfort; however, their effectiveness and acceptability may vary. Cryotherapy has emerged as a simple, inexpensive, and non-pharmacological approach for pain control, but evidence regarding its use as a topical anesthetic alternative in pediatric dentistry remains limited.

This randomized controlled clinical trial aimed to compare the effects of cryotherapy and conventional topical anesthetic agents on injection-related pain and the overall treatment experience in children. A total of 100 healthy children aged 7-10 years requiring maxillary buccal infiltration anesthesia for dental treatment were enrolled. Eligible participants had no previous experience with dental treatment under local anesthesia, demonstrated positive behavior ratings (Frankl scores 3-4), and had Modified Dental Anxiety Scale scores below 19. Participants were randomly allocated to one of four groups (n = 25 each): 10% lidocaine spray, 20% benzocaine gel, cryotherapy with distilled water cooled to +4°C, or cooled 10% lidocaine spray stored at +4°C.

The primary outcome was injection-related pain assessed using the Wong-Baker Pain Rating Scale. Secondary outcomes included dental anxiety, treatment compliance, taste acceptability, and physiological responses measured by pulse rate and oxygen saturation before and during treatment. Behavioral and anxiety assessments were performed using validated pediatric scales.

Ethical approval was obtained from the Ondokuz Mayıs University Clinical Research Ethics Committee (Approval No. 2024/316). The study was also approved by the Turkish Medicines and Medical Devices Agency (TMMDA; Approval No. E-24931227-514.04.02-5719207) and registered at ClinicalTrials.gov (NCT07198022). The study was designed to determine whether cryotherapy could serve as a safe and effective alternative to conventional topical anesthetic agents for improving the pediatric dental treatment experience.

**General Information**

**Protocol Title**

Pain, Sensory Acceptability, and Treatment Compliance Following Cryotherapy and Topical Anesthetics in Children: A Randomized Clinical Trial
**Clinical Trial Registration**

ClinicalTrials.gov Identifier: NCT07198022

Registration submitted: 25/09/2025

Registration posted: 30/09/2025

**Sponsor/Funder**

This investigator-initiated academic study was conducted at the Department of Pediatric Dentistry, Faculty of Dentistry, Ondokuz Mayıs University, Turkey. No external funding was received for the conduct of the study.

**Investigators and Responsibilities**

**Emine Zehra Aydın Çetin, DDS**
Researcher
Department of Pediatric Dentistry, Faculty of Dentistry, Ondokuz Mayıs University, Samsun, Turkey

**Responsibilities:**

- Participant recruitment and screening
- Clinical implementation of study procedures
- Randomization implementation
- Data collection and management
- Literature review
- Drafting of the thesis and manuscript
- Interpretation of findings

**Bilal Özmen, DDS, PhD, Associate Professor**
Principal Investigator
Department of Pediatric Dentistry, Faculty of Dentistry, Ondokuz Mayıs University, Samsun, Turkey

**Responsibilities:**

- Study conception and supervision
- Methodological guidance
- Statistical analysis
- Critical revision of the manuscript
- Final approval of the study report and manuscript

**Research Site**

Department of Pediatric Dentistry
Faculty of Dentistry
Ondokuz Mayıs University
Kurupelit Campus, Samsun, Turkey

Telephone: +90 362 312 19 19

**Clinical and Technical Facilities Involved**

All clinical procedures, participant assessments, data collection, and follow-up evaluations were performed at the Department of Pediatric Dentistry, Faculty of Dentistry, Ondokuz Mayıs University, Samsun, Turkey.

No external clinical laboratories were involved in the study. Physiological measurements, including pulse rate and oxygen saturation, were obtained chairside using a pulse oximeter within the clinical setting.

**Rationale and Background Information**

Pain associated with local anesthetic injections remains one of the primary sources of fear and anxiety in pediatric dental patients. (1) Needle-related fear may negatively influence treatment compliance, increase behavioral management difficulties, and contribute to avoidance of future dental care. (2,3) As a result, reducing discomfort during local anesthetic administration is an important component of pediatric dental practice. (4)

Topical anesthetic agents are routinely applied before local anesthetic injections to minimize pain caused by needle penetration. (4) Among the most commonly used agents are lidocaine sprays and benzocaine gels. (2,4) Despite the widespread use of topical anesthetic agents, the search for alternative or adjunctive methods to enhance injection comfort in pediatric patients continues.

Cryotherapy, defined as the local application of cold to tissues, has long been used for pain control in medicine because of its analgesic and anti-inflammatory effects. (5,6) Cooling reduces nerve conduction velocity and may temporarily decrease pain perception at the application site. (7) As a simple, inexpensive, and non-pharmacological intervention with minimal adverse effects, cryotherapy has attracted increasing interest as a potential alternative to conventional topical anesthetic agents. (8,9)

Several clinical studies have investigated the effectiveness of cryotherapy during local anesthetic administration in children. (10-12) Previous randomized clinical trials have reported reduced pain perception and improved patient comfort when cryotherapy was applied before dental injections. (10-12)

In dental settings, pediatric patients commonly exhibit a natural avoidance of materials that induce unpleasant sensory experiences, such as pain, burning sensations, or unfavorable taste. These sensory perceptions may significantly influence children's acceptance of dental procedures and their willingness to cooperate during treatment [13]. Children's behavioral responses and treatment compliance are equally important clinical outcomes alongside pain reduction, yet the impact of topical anesthetic agents and cryotherapy on these dimensions remains insufficiently investigated. Despite the widespread use of these agents in pediatric dentistry, no study to date has systematically evaluated taste acceptability and treatment compliance alongside pain outcomes in a comparative four-arm design. Therefore, this study aimed to evaluate the effects of cryotherapy compared with routinely used topical anesthetic agents on injection-related pain, taste acceptability, and treatment compliance in pediatric patients, alongside dental anxiety and physiological responses, to provide a more comprehensive assessment of the pediatric patient experience extending beyond pain measurement to incorporate sensory and behavioral dimensions.

**Study Goals and Objectives**

**Study Goal**

The overall goal of this study was to evaluate the effectiveness of cryotherapy as an alternative to routinely used topical anesthetic agents during local anesthetic administration in pediatric dental patients.

**Primary Objective**

To compare the effects of cryotherapy, cooled topical anesthetic spray, topical anesthetic spray, and topical anesthetic gel on injection-related pain during maxillary buccal infiltration anesthesia in children aged 7-10 years.

**Secondary Objectives**

- To evaluate the effects of the interventions on dental anxiety.
- To assess treatment compliance during dental treatment.
- To compare taste acceptability among the tested interventions.
- To evaluate changes in physiological parameters, including pulse rate and oxygen saturation, during treatment.
- To determine whether cryotherapy may serve as a practical non-pharmacological alternative to conventional topical anesthetic agents in pediatric dentistry.

**Study Design**

This study was designed as a prospective, randomized, controlled, parallel-group clinical trial conducted at the Department of Pediatric Dentistry, Faculty of Dentistry, Ondokuz Mayıs University, Turkey.

The study evaluated four interventions applied prior to local anesthetic administration: (1) 10% lidocaine spray, (2) 20% benzocaine gel, (3) cryotherapy using distilled water cooled to +4°C, and (4) cooled 10% lidocaine spray stored at +4°C.

A total of 100 children aged 7-10 years who required maxillary buccal infiltration anesthesia for dental treatment were recruited. Participants were randomly assigned to one of four intervention groups (n = 25 per group) using sequentially numbered opaque sealed envelopes prepared by an independent researcher.

The study was conducted using a single-blinded, assessor-blinded design. Participants were blinded to group allocation, while outcome assessments were performed by an independent evaluator who was unaware of the intervention received by each participant. Operator blinding was not feasible because of the distinct physical characteristics of the tested materials.

The expected study duration extended from participant recruitment to completion of data collection and statistical analysis. The primary outcome measure was injection-related pain assessed using the Wong-Baker FACES Pain Rating Scale. Secondary outcomes included dental anxiety, treatment compliance, taste acceptability, pulse rate, and oxygen saturation.

Participant recruitment and clinical data collection were carried out between 25/09/2025 and 15/01/2026 Department of Pediatric Dentistry, Faculty of Dentistry, Ondokuz Mayıs University, Turkey. Eligible participants were enrolled consecutively during this period until the target sample size was achieved.

The anticipated duration of participant involvement was limited to a single treatment visit, including baseline assessment, topical anesthetic application, local anesthetic administration, and outcome evaluation.

**Study Population**

The study population consisted of pediatric patients attending the Department of Pediatric Dentistry, Faculty of Dentistry, Ondokuz Mayıs University, Turkey, who required maxillary buccal infiltration anesthesia as part of their dental treatment. Participant recruitment and clinical data collection were conducted between 25/09/2025 and 15/01/2026.

Children aged 7-10 years who met the eligibility criteria were consecutively screened and invited to participate. Written informed consent was obtained from the parents or legal guardians of all participants prior to enrollment.

**Eligibility Criteria**

**Inclusion Criteria**

- Children aged 7-10 years.
- Ability to read and understand the assessment scales used in the study.
- American Society of Anesthesiologists (ASA) physical status I or II.
- Positive behavior according to the Frankl Behavior Rating Scale (scores 3-4).
- Modified Dental Anxiety Scale (MDAS) score < 19.
- Requirement for treatment of primary teeth under maxillary buccal infiltration anesthesia.
- No previous dental treatment experience.
- No known allergy to the topical anesthetic agents used in the study.

**Exclusion Criteria**

- Age below 7 years or above 10 years.
- Inability to understand or complete the assessment scales.
- ASA physical status > II.
- Negative behavior according to the Frankl Behavior Rating Scale (scores 1-2).
- MDAS score ≥ 19.
- Previous dental treatment experience.
- Known allergy or hypersensitivity to any study material.
- Refusal of parental consent or child assent.

**Withdrawal Criteria**

- Parent/guardian request
- Child refusal to continue
- Development of an adverse reaction
- Inability to complete study procedures.

**Figure 1**. Study Flow Diagram

**Sample Size Calculation**

Sample size calculation was performed using G*Power software (version 3.1.9.7). The effect size (Cohen's f = 0.50) was estimated based on pain perception outcomes reported by Dhingra et al. (2022) (10). Assuming a significance level of α = 0.05 and a statistical power of 95%, the minimum required sample size was calculated as 76 participants (19 participants per group). To compensate for potential dropouts and increase statistical reliability, the sample size was increased to 100 participants, resulting in 25 participants in each study group.

**Randomization and Allocation Concealment**

Eligible participants were randomly assigned to one of four study groups using a random allocation sequence generated by an independent researcher. Allocation concealment was ensured through the use of sequentially numbered opaque sealed envelopes. Each envelope contained the intervention assignment and was opened only after participant enrollment.

Participants were allocated in a 1:1:1:1 ratio to one of the following groups:

- Group 1: 10% lidocaine spray
- Group 2: 20% benzocaine gel
- Group 3: Cryotherapy using distilled water cooled to +4°C
- Group 4: Cooled 10% lidocaine spray stored at +4°C

**Blinding**

Due to the distinct physical characteristics of the study materials, operator blinding was not feasible. Therefore, the study was conducted as a single-blinded randomized clinical trial in which participants were unaware of group allocation.

Outcome assessments were performed by an independent evaluator who was blinded to the intervention groups. This assessor-blinded approach was adopted to minimize assessment bias and improve the validity of outcome measurements.

**Methodology**

All study procedures were performed at the Department of Pediatric Dentistry, Faculty of Dentistry, Ondokuz Mayıs University, Turkey, according to a standardized protocol. Following eligibility assessment, informed consent procedures, and baseline evaluations, participants were randomly assigned to one of four intervention groups. Topical anesthetic application or cryotherapy was performed before local anesthetic administration, after which outcome assessments were completed according to the predefined study protocol.

The methodology was designed to ensure standardized intervention delivery, outcome assessment, and data collection across all study groups.

**Interventions**

Participants were randomly allocated to one of four intervention groups prior to local anesthetic administration:

**Group 1: Topical Anesthetic Spray**

A 10% lidocaine hydrochloride topical spray (Locanest®, Avixa İlaç San. ve Tic. Ltd. Şti., Turkey) was applied to the dried mucosal surface at the injection site.

**Group 2: Topical Anesthetic Gel**

A 20% benzocaine topical gel with strawberry flavor (Vision®, Anadolu Diş Deposu San. ve Tic. A.Ş., Turkey) was applied to the dried mucosal surface at the injection site.

**Group 3: Cryotherapy**

Cryotherapy was performed using distilled water stored at +4°C. The cooled distilled water was applied immediately after removal from refrigeration.

**Group 4: Cooled Topical Anesthetic Spray**

A 10% lidocaine hydrochloride topical spray (Locanest®, Avixa İlaç San. ve Tic. Ltd. Şti., Turkey) stored at +4°C prior to application was used.

For all groups, the intervention was applied to the dried mucosal surface using a cotton pellet and gently maintained at the injection site for 20 seconds. Following application, participants were asked whether they perceived numbness at the application site.

Subsequently, buccal infiltration anesthesia was administered in the maxillary molar region by a single operator using 4% articaine with epinephrine 6 mcg/m (Ultracaine®, Sanofi Aventis Pharmaceuticals Ltd., Turkey). Standardized injection procedures were used for all participants.

**Study Procedures and Timing of Assessments**

1. Potential participants attending the Department of Pediatric Dentistry were screened for eligibility according to the predefined inclusion and exclusion criteria.
2. Written informed consent was obtained from the parents or legal guardians of eligible children prior to enrollment.
3. Baseline demographic information, including age and sex, was recorded.
4. Pre-procedural dental anxiety was assessed using the Modified Dental Anxiety Scale (MDAS) and the Facial Image Scale (FIS).
5. Baseline physiological measurements, including pulse rate and oxygen saturation (SpO₂), were recorded using a pulse oximeter.
6. Participants were randomly assigned to one of the four intervention groups.
7. The assigned topical anesthetic agent or cryotherapy intervention was applied to the dried mucosal surface at the injection site using a cotton pellet for 20 seconds.
8. Physiological measurements (pulse rate and oxygen saturation) were recorded during topical application.
9. Buccal infiltration anesthesia was administered in the maxillary molar region using 4% articaine with epinephrine 1:100,000 by a single operator.
10. Physiological measurements (pulse rate and oxygen saturation) were recorded during needle penetration.
11. Following completion of the injection procedure, pain perception was assessed using the Wong-Baker FACES Pain Rating Scale (WBPRS).
12. Taste acceptability was evaluated using the Three-Point Hedonic Scale.
13. Treatment compliance was assessed using the Frankl Behavior Rating Scale.
14. All outcome measurements were completed during the same clinical visit.

**Outcome Measures**

**Primary Outcome**

The primary outcome of the study was injection-related pain associated with local anesthetic administration. Pain perception was assessed immediately after the injection procedure using the Wong-Baker FACES Pain Rating Scale (WBPRS), a validated self-report measure ranging from 0 (no pain) to 10 (worst pain).

**Secondary Outcomes**

**Dental Anxiety**

Dental anxiety was assessed before the intervention using the Modified Dental Anxiety Scale (MDAS) and the Facial Image Scale (FIS). These instruments were used to evaluate participants' baseline anxiety levels prior to local anesthetic administration.

**Treatment Compliance**

Treatment compliance was assessed using the Frankl Behavior Rating Scale. Behavioral responses during treatment were classified according to the four-point Frankl scale, ranging from definitely negative to definitely positive behavior.

**Taste Acceptability**

Taste acceptability of the applied intervention was assessed immediately after application using a Three-Point Hedonic Scale. Participants were asked to rate their sensory acceptance of the intervention as unpleasant, neutral, or pleasant.

**Physiological Parameters**

Physiological responses were assessed by measuring pulse rate and oxygen saturation (SpO₂) using a pulse oximeter. Measurements were obtained at three time points:

- Before intervention (baseline)
- During topical application
- During local anesthetic injection

These measurements were used to evaluate physiological changes associated with anxiety and procedural stress.

**Safety Considerations**

The interventions evaluated in this study involved routinely used topical anesthetic agents and cryotherapy, both of which are considered low-risk procedures in pediatric dental practice.

Before enrollment, participants were screened for eligibility, including assessment of medical history and known allergies to topical anesthetic agents. Children with known hypersensitivity to lidocaine, benzocaine, or any component of the study materials were excluded from participation.

All procedures were performed by trained personnel under standard clinical conditions at the Department of Pediatric Dentistry, Ondokuz Mayıs University. Participants were continuously monitored throughout the procedure. Pulse rate and oxygen saturation measurements were obtained during treatment to monitor physiological responses.

Any unexpected adverse event, allergic reaction, or participant discomfort would have resulted in immediate discontinuation of the intervention and provision of appropriate clinical management. No invasive procedures beyond routine dental treatment were performed as part of the study.

**Follow-up**

Participant involvement was limited to a single clinical visit. All interventions, outcome assessments, and physiological measurements were completed during the same appointment.

Because the study evaluated immediate responses to topical anesthetic agents and cryotherapy during local anesthetic administration, no additional follow-up visits were required. Participants continued to receive routine dental care according to standard clinical practice.

**Data Management and Statistical Analysis**

All study data were recorded using standardized data collection forms and subsequently entered into an electronic database for analysis. Data accuracy was verified prior to statistical evaluation. Participant confidentiality was maintained throughout the study by using coded identifiers instead of personal information.

Sample size calculation was performed using G*Power software (version 3.1.9.7). The effect size was estimated based on previously published data, assuming a significance level of α = 0.05 and a statistical power of 95%.

Statistical analyses were performed using IBM SPSS Statistics software. Descriptive statistics were calculated for all study variables. Continuous variables were summarized using means, standard deviations, medians, minimum values, and maximum values as appropriate.

Repeated-measures analysis of variance (ANOVA) was used to evaluate changes in pulse rate and oxygen saturation over time, with study group and sex included as factors. Tukey's Honestly Significant Difference (HSD) test was applied for post hoc comparisons when appropriate.

For non-parametric outcome variables, Mann-Whitney U tests were used for two-group comparisons and Kruskal-Wallis tests were used for comparisons among multiple groups.

A p-value of less than 0.05 was considered statistically significant.

**Quality Assurance**

All study procedures were conducted according to a standardized protocol to ensure consistency across participants. Clinical procedures, outcome assessments, and data collection methods were standardized before study initiation. All measurements were obtained using validated assessment instruments and calibrated equipment. Data were reviewed for completeness and accuracy prior to statistical analysis.

**Expected Outcomes of the Study**

The study aimed to provide evidence regarding the effectiveness of cryotherapy and cooled topical anesthetic agents in reducing injection-related pain in pediatric dental patients. In addition to pain perception, the study aimed to improve understanding of the effects of these interventions on dental anxiety, physiological responses, taste acceptability, and treatment compliance.

The findings may contribute to the development of simple, safe, and patient-friendly approaches for improving children's experiences during local anesthetic administration and to support evidence-based decision-making in pediatric dental practice.

**Dissemination of Results and Publication Policy**

The findings of the study will be disseminated through publication in peer-reviewed scientific journals and presentation at national and international scientific meetings. Authorship will be determined according to internationally accepted authorship criteria. Study results may also be used for academic and educational purposes while maintaining participant confidentiality.

**Duration of the Project**

Protocol development and ethical approval procedures were completed before study initiation. Participant recruitment and data collection were conducted between September 2025 and January 2026. Data analysis and manuscript preparation were performed following completion of data collection.

**Protocol Amendments and Deviations**

The original trial protocol was written in Turkish and approved by the Ondokuz Mayis University Clinical Research Ethics Committee (Approval No. 2024/316; 31/07/2024) and by the Turkish Medicines and Medical Devices Agency (Approval No. E-24931227-514.04.02-5719207; 28/08/2025). The present English-language protocol corresponds to that approved plan. The following changes and deviations from the original protocol are reported for transparency.

Timing of the study. The original protocol specified a planned study period of 01/04/2025 to 02/01/2026. Because of delays during the ethics and regulatory review process, participant enrolment could not begin on the originally planned date. Actual recruitment and data collection were therefore carried out between 25/09/2025 and 15/01/2026. This deviation affected only the timing of the study; the eligibility criteria, interventions, group allocation, and outcome measures were not changed.

Statistical analysis. The original protocol specified the assessment instruments and study outcomes but did not detail the specific statistical tests to be applied. The statistical analysis plan was subsequently specified in full, on the basis of the pre-defined outcomes and before data analysis, as described in the Data Management and Statistical Analysis section. No changes were made to the planned outcomes, and the analysis followed the outcomes defined in the original protocol.

No other deviations from the original protocol occurred. There were no changes to the eligibility criteria, sample size, randomisation procedure, interventions, or primary and secondary outcome measures after the start of participant enrolment.

**Project Management**

The study was conducted at the Department of Pediatric Dentistry, Faculty of Dentistry, Ondokuz Mayıs University, Turkey.

Emine Zehra Aydın Çetin was responsible for participant recruitment, clinical implementation of study procedures, data collection, data management, and manuscript preparation.

Bilal Özmen was responsible for study conception, supervision, methodological guidance, statistical oversight, and critical revision of the manuscript.

**Problems Anticipated**

Several potential limitations were anticipated prior to study initiation. First, the study was conducted at a single center, which may limit the generalizability of the findings to other clinical settings and populations. Second, the primary and several secondary outcome measures, including pain perception, taste acceptability, and dental anxiety, relied on self-report scales, which are inherently subject to individual variability and developmental differences in children's ability to accurately communicate their experiences. Third, the study was restricted to a specific age group (7-10 years) and a single injection technique (maxillary buccal infiltration), which may limit applicability to younger children or other anesthetic procedures. Fourth, inclusion was limited to children with positive baseline behavior ratings, which may have reduced the variability of behavioral outcomes and limited conclusions regarding children with higher levels of dental anxiety or uncooperative behavior.

**Ethics**

The study was conducted in accordance with the ethical principles outlined in the Declaration of Helsinki and applicable national regulations governing clinical research involving human participants.

Ethical approval was obtained from the Ondokuz Mayıs University Clinical Research Ethics Committee (Approval No. 2024/316; 31/07/2024) prior to study initiation. In addition, regulatory approval was granted by the Turkish Medicines and Medical Devices Agency (TMMDA) (Approval No. E-24931227-514.04.02-5719207).

Participation in the study was entirely voluntary. Before enrollment, parents or legal guardians of all eligible children received detailed verbal and written information regarding the study objectives, procedures, potential risks, and expected benefits. Written informed consent was obtained from parents or legal guardians prior to participation. Children were also provided with age-appropriate information regarding the study and their willingness to participate was respected throughout the research process.

Participant confidentiality was protected throughout the study. Personal identifiers were removed from study records and replaced with coded identification numbers. Access to study data was restricted to the research team and all data were stored securely in accordance with institutional policies.

Participants were free to withdraw from the study at any time without affecting their access to dental treatment or any future care provided by the institution.

**Informed Consent Forms**

Copies of the informed consent forms, including the Child Assent Form and the Parental İnformed Consent Form, both available in Turkish, are available upon request.

**References**

1. Siddiqui TM, Wali A, Abdullah H, Khan FA, Tanvir R, Siddiqui MR. Evaluation of fear of injections and its association with avoidance of dental treatment. J Res Dent 2016;4:81-85.
2. Alvarez GG, Romero CC, Israel G et al. Topical anesthetics in pediatric dentistry: A literature Review. Int. J. Appl. Dent. 2022; 8(3): 283-286.
3. Yon MJY, Chen KJ, Gao SS, Duangthip D, Lo ECM, Chu CH. An introduction to assessing dental fear and anxiety in children. Healthcare (Basel) 2020;8(2):86.
4. Lee HS. Recent advances in topical anesthesia. J Dent Anesth Pain Med.2016;16(4):237-244.
5. Kwiecien SY, McHugh MP. The cold truth: the role of cryotherapy in the treatment of injury and recovery from exercise. Eur J Appl Physiol. 2021.
6. Bleakley CM, Davison GW. Cryotherapy and inflammation: evidence beyond the cardinal signs. Phys Ther Rev 2010;15(6):430-435.
7. Algafly AA, George KP. The effect of cryotherapy on nerve conduction velocity, pain threshold and pain tolerance. Br J Sports Med. 2007;41(6):365-9.
8. Amrollahi N, Rastghalam N, Faghihian R. Effect of pre-cooling on pain associated with dental injections in children: a systematic review. J Evidence-based Dent Pract. 2021;21(3):101588.
9. Saeedi P, Shirazi AS, Ghorbani M. The effectiveness of cryotherapy and cooled topical anesthesia compared with conventional topical anesthesia in alleviating intraoral injection pain: A systematic review and meta-analysis. Int J Paediatr Den. 2025;(35):655-674.
10. Dhingra N, Mathur S, Malik M. Effectiveness of cryotherapy, sucrose solution and a combination therapy for pain control during local anesthesia in children: a split mouth study. J Clin Pediatr Dent 2022:46(6);1-5.
11. Chilakamuri S, Svsg N, Nuvvula S. The effect of pre-cooling versus topical anesthesia on pain perception during palatal injections in children aged 7-9 years: a randomized split-mouth crossover clinical trial. J Dent Anesth Pain Med 2020;20(6):377-386.
12. Maganur PC, Ghawi AA, Arishi GD, Bahammam HA, Alessa N, Hamed NE, Jawhali NA, Sawady M, Manqari AIH, Vishwanathaiah S. A comparative study on pain perception in children, after application of pre-cooled and plain topical anaesthetic gel during local anaesthetic administration-A parallel three-arm randomised control trial. Children (Basel) 2025;12(7):863.
13. Mohan R, Madiraju GS, Chieng CY, Almugla YM, Asiri FYI. Evaluation of taste acceptance of three different fluoride varnishes in children with autistic spectrum disorder: a randomized clinical trial. J Clin Me*.* 2025;14(6):1948.
