# Supplementary material for: Pain, sensory acceptability, and treatment compliance following cryotherapy and topical anesthetics in children: A randomized clinical trial
Source: PLoS One. 2026 Nov 3;21(11):e0362074. doi: 10.1371/journal.pone.0362074 (PMC13730363; doi:10.1371/journal.pone.0362074)
Supplement: Supplementary file 5 [file pone.0362074.s008.docx]

25^th^ September, 2026

**Dear Editor Dr. Aline Castilho and Reviewers,**

We sincerely thank the Academic Editor and the reviewers for their time and for their careful and constructive evaluation of our manuscript. We have addressed each comment point by point below. The reviewers' comments are shown in italics, followed by our responses.

**Journal Requirements**

1. **Please ensure that your manuscript meets PLOS ONE's style requirements, including those for file naming.**

The manuscript has been revised according to the PLOS ONE style templates, and all files have been named in accordance with the journal's requirements.

**2. We are unable to open your Supporting Information file [S1.Data Set.sav].**
The dataset has been re-uploaded in .xlsx format (S1_Dataset.xlsx), together with a data dictionary.

**3. Please include captions for your Supporting Information files at the end of your manuscript, and update any in-text citations to match accordingly.**
Captions for the Supporting Information files (S1 Dataset and S1 Table) have been added at the end of the manuscript, and the in-text citations have been updated accordingly.

**4. If the reviewer comments include a recommendation to cite specific previously published works, please review and evaluate these publications to determine whether they are relevant and should be cited. There is no requirement to cite these works unless the editor has indicated otherwise.**

The reviewers did not recommend citing specific previously published works.

**Reviewer #1:**

1- ***Specify the statistical test and model assumptions used in the sample size calculation so that the reported effect size (Cohen's f = 0.50), power, and required sample size can be independently evaluated and linked to the planned primary analysis.***

-The sample size calculation has been described in more detail (lines 117-120). Although the primary outcome was analysed with the non-parametric Kruskal-Wallis test, the one-way ANOVA framework was used for the calculation, and the enrolled sample (n = 100) exceeded the calculated requirement by 24 participants, providing a margin for the lower efficiency of the non-parametric test. The limitation related to the assumed large effect size has been addressed in the Limitations section (lines 439-442).

- ***2- Clarify whether nonsignificant interaction terms were removed from the repeated-measures ANOVA models and whether reduced models containing only main effects were refit before conducting inference on treatment, sex, and time effects.***
- ***12- Clarify whether the repeated-measures ANOVA results presented in the manuscript arise from the full factorial model or from a reduced model after evaluation of interaction terms.***

-We thank the reviewer for these important comments. In the original submission, the repeated-measures ANOVA results were derived from the full factorial model, including group, sex, and measurement period and all their interactions. As none of the sex-related effects or interactions were significant, and sex was not a factor of interest in the study objectives, sex was removed from the model. The pulse rate and oxygen saturation data were reanalysed using a repeated-measures ANOVA with measurement period as the within-subject factor and group as the between-subject factor. The group x measurement period interaction was retained, as it directly addresses whether changes over time differed among the interventions. The revised results are presented in the Results section, lines 220-243 and Tables 2 and 3.

***3- Reanalyze the pulse-rate and oxygen-saturation outcomes using reduced models if all interaction terms were nonsignificant, and report the resulting main-effect estimates and p-values from those reduced models.***

-The group x time interaction was retained, as it constitutes the primary test of whether changes over time differed among the interventions; moreover, in repeated-measures ANOVA, the between-subject test of group is based on each participant's mean across time points and is therefore unaffected by this term. The main-effect estimates, effect sizes, and p-values from the revised model are reported in the Results section, lines 220-243 and Tables 2 and 3.

***4- Revise the interpretation of the pulse-rate findings because a nonsignificant group-by-time interaction does not support a conclusion that pulse-rate changes over time differed among treatment groups.***

-We agree that the nonsignificant group x time interaction does not support differential changes in pulse rate among the groups. The statement has been removed from the Abstract and Results, and the corresponding paragraph in the Discussion has been revised to state that pulse rate increased over time in all groups without significant differences among the interventions (lines 337-343).

***5- Report assessment of the sphericity assumption for repeated-measures ANOVA and, if violated, provide the corresponding Greenhouse-Geisser or Huynh-Feldt corrected results.***

***-***Mauchly's test indicated a violation of sphericity for both pulse rate (χ²(2) = 8.10, p = 0.017) and oxygen saturation (χ²(2) = 12.90, p = 0.002). Accordingly, Greenhouse-Geisser corrected results are now reported in the Results section (lines 223-224, 237-238).

***6- Report effect-size measures for repeated-measures ANOVA results, such as partial eta-squared (ηp²), to facilitate interpretation of both significant and nonsignificant findings.***

-As suggested, partial eta-squared (ηp²) values are now reported for all main effects and interactions of the repeated-measures ANOVA (lines 223-224, 237-238).

***7- Provide confidence intervals for treatment-group comparisons and treatment effects rather than relying primarily on p-values and descriptive statistics.***

-As recommended, 95% confidence intervals are now reported for between-group differences in pulse rate and oxygen saturation (S1 Table) and for group means of all ordinal outcomes (Table 4).

***8- Avoid interpreting nonsignificant superiority tests as evidence that treatments are equivalent, comparable, or noninferior unless a formal equivalence or noninferiority framework was prespecified and analyzed.***

-As the study was not designed as an equivalence or non-inferiority trial, expressions implying equivalence have been revised throughout the Abstract, Discussion, and Conclusions to state that the outcomes did not differ significantly among the groups. In addition, this has been acknowledged in the Limitations section.

***9- Clarify whether post hoc comparisons were performed following the significant overall time effect observed for pulse rate and identify which measurement periods differed statistically.***

-Bonferroni-adjusted pairwise comparisons showed that pulse rate increased significantly only from the pre-operative to the post-operative period (p = 0.012). As the time effect was not significant for oxygen saturation, post hoc comparisons were not indicated for this outcome.

***10- Discuss the potential impact of multiple secondary outcomes and repeated hypothesis testing on the overall type I error rate.***

-We agree that testing multiple secondary outcomes without adjustment increases the risk of type I error. Secondary outcomes are now described as exploratory in the Methods, and this limitation has been acknowledged in the Limitations section (lines 444-446).

***11- Discuss the potential ceiling effect introduced by restricting eligibility to children with positive baseline FRANKL scores (3-4), as this may have limited the ability to detect clinically meaningful differences in treatment compliance.***

-We agree that including only children with FRANKL scores of 3-4 may have produced a ceiling effect and limited the detection of differences in compliance, and that the findings may not apply to children with negative baseline behavior. This has been acknowledged in the Limitations section (lines 457-460).

***13- Acknowledge that the sample size calculation was based on a large assumed effect size (Cohen's f = 0.50), which may have limited power to detect smaller but potentially clinically meaningful between-group differences.***

-We acknowledge that the large assumed effect size (Cohen's f = 0.50) may have limited the power to detect smaller but clinically meaningful differences, particularly in pain perception, although the enrolled sample (n = 100) exceeded the calculated requirement (n = 76) with no loss to follow-up. This has been added to the Limitations section (lines 437-441).

***14- Report the percentage corresponding to each sex count in Table 2, in addition to the raw counts.***

-As suggested, the percentages corresponding to each sex count have been added to Table 1 (formerly Table 2).

***15- Replace the minimum-maximum range in Table 5 with the interquartile range (IQR), as the IQR is a more appropriate measure of dispersion for ordinal variables summarized by the median.***

-We thank the reviewer for this suggestion. The minimum-maximum ranges in the former Table 5 (now Table 4) have been replaced with interquartile ranges, and the table has been regenerated directly from the dataset.

**Reviewer #2:** 
***1)	Perhaps it could be added to the introduction that local anesthetic formulations have an acidic pH, which can cause discomfort during injection.***

-Thank you for this recommendation. A sentence on the acidic pH of local anesthetic solutions and its contribution to injection discomfort has been added to the Introduction (lines 67-70) as reference [9].

***2)	Furthermore, I find it interesting that the results of cryotherapy are similar to those of other groups, which could make the procedure simpler and less expansive.***

-We thank the reviewer for highlighting this clinically relevant point. A sentence emphasizing the practical advantages of cryotherapy, including its low cost, ready availability, and absence of pharmacological side effects, has been added to the Discussion (lines 381,384). This aspect is also reflected in the Conclusions section.

***3)	I suggest replacing Table 1 with a graph. I believe this would improve the presentation of the results.***

***-***Thank you for this suggestion. As Table 1 contained only the manufacturer and composition information of the materials, which duplicated the Methods section, it has been removed in line with Reviewer #3's comment.

**Reviewer #3:**

***o Table 1 presents redundant information (such as "10% lidocaine hydrochloride spray", "Locanest®", etc.) that is already clearly and fluently detailed in the Methods text (lines 144–151). Removing the table or integrating its content into the narrative text will streamline the manuscript and avoid unnecessary repetition.***

***-***As suggested, Table 1 has been removed, since its content is now described in the Methods section. The remaining tables have been renumbered accordingly.

***o Established literature in Pediatric Dentistry and Dental Anesthesiology indicates that topical anesthetics (such as benzocaine and lidocaine) require 1 to 2 minutes of mucosal contact to achieve adequate tissue penetration and clinical efficacy (2 to 3 mm). Applying them for only 20 seconds may have resulted in sub-optimal superficial anesthesia, potentially explaining the lack of significant differences between the pharmacological groups and cooled water. The authors should justify and reference this methodological choice.***

-We agree that the 20-second application time is shorter than the recommended 1-2 minutes and may have resulted in suboptimal mucosal anesthesia. This duration was kept identical across all groups for standardization, and injection was performed only after the child confirmed perceived numbness. This has been acknowledged in the Limitations section (lines 452-454).

o ***The manuscript displays a common misconception by directly mirroring inclusion criteria into the exclusion criteria (e.g., stating age range in inclusion and the opposite in exclusion). Exclusion criteria should specify conditions in participants who meet the inclusion criteria but possess an additional factor that precludes participation (e.g., presence of acute pain, recent analgesic use, local anatomical alterations). A revision of this section is recommended.***

-The eligibility criteria have been revised so that the exclusion criteria no longer mirror the inclusion criteria and instead specify conditions that precluded participation among otherwise eligible children (lines 123-131).

**o In the PDF version of the manuscript (pages 12–13, lines 224–233), there is a formatting glitch that compromises the clarity of parts of Table 3, making several headers and interactions (Group x Gender x Measurement) illegible. The table must be replaced with a clear, editable, high-resolution version.**

-We thank the reviewer for pointing out this issue. Following the reanalysis requested by Reviewer #1, the pulse rate and oxygen saturation tables (now Tables 2 and 3) have been regenerated from the dataset as simplified, editable tables, with the statistical results reported in the text.

**o Although these terms are sometimes used interchangeably in clinical practice, consistent terminology is required in scientific writing. Since measurements were obtained using a pulse oximeter (line 173), the term "pulse rate" should be used consistently throughout the manuscript.**

-As measurements were obtained using a pulse oximeter, the term "pulse rate" is now used consistently throughout the manuscript.

**o Injection pain perception is directly influenced by needle gauge, length, and the volume/rate of anesthetic solution administration. Although the agent was specified (4% articaine with epinephrine), the manuscript omits the volume administered and needle specifications (e.g., 27G or 30G; short or extra-short). Providing these details is essential for study reproducibility. It would be better if the authors included the epinephrine concentration (such as 1:100,000 or 1:200,000), as it is more commonly reported.**

-The epinephrine concentration, the syringe and needle specifications, and the volume of anesthetic solution administered (approximately 1.5 mL) have been added to the Methods section, (lines 157-160 ).

**o A patient's baseline symptomatic status (e.g., asymptomatic tooth vs. irreversible pulpitis or active pain) significantly alters pain threshold and pre-procedural anxiety. The manuscript merely states "indication for treatment of primary teeth" (line 124). It is critical to specify the clinical diagnoses included and discuss their potential confounding effect on pain perception and anxiety outcomes.**

- All included teeth were clinically asymptomatic, and the indicated treatments (restorative treatment, pulpotomy, or extraction of primary maxillary molars) have been specified in the Methods (lines 129-131). The potential confounding effect of baseline symptomatic status has been discussed (lines 305-312); as pain and compliance were assessed during injection, before any treatment was initiated, this effect was minimized.

**o The study employed a 4-arm parallel group design (25 different children per group). In pediatric pain and anxiety research, split-mouth or cross-over designs are often preferred as participants serve as their own controls, thereby minimizing inter-individual variability. Inherent individual differences in pain thresholds among participants may have masked significant between-group differences. This limitation should be explicitly addressed in the Limitations section (line 413).**

- We agree that split-mouth and crossover designs reduce inter-individual variability; however, they were not feasible here, as a second intervention would have introduced a carry-over effect in children without previous dental experience and compromised participant blinding. We acknowledge that inter-individual differences in pain thresholds may have masked between-group differences, and this has been added to the Limitations section (lines 446-448).

**Reviewer #4:**

***Interesting subject matter. The study design I feel is well done, especially considering the age of the population that was assessed. Cryotherapy is definitely a novel method to use with children, but provides simple and effective methodology.***

-We sincerely thank the reviewer for the positive evaluation of our study and for recognizing the novelty and practicality of cryotherapy in pediatric patients.

Dr. Emine Zehra AYDIN ÇETİN

Corresponding Author
