# Supplementary material for: Pain, sensory acceptability, and treatment compliance following cryotherapy and topical anesthetics in children: A randomized clinical trial
Source: PLoS One. 2026 Nov 3;21(11):e0362074. doi: 10.1371/journal.pone.0362074 (PMC13730363; doi:10.1371/journal.pone.0362074)
Supplement: Supplementary file 6 [file pone.0362074.s009.docx]

14^th^ October, 2026

**Dear Editor Dr. Aline Castilho and Reviewer,**

We sincerely thank the Academic Editor and the reviewer for their time and for their careful and constructive evaluation of our manuscript. We have addressed each comment point by point below. The reviewer’s comments are shown in **bold**, followed by our responses.

**Journal requirements:** The reference list was checked; no retracted articles are cited.

**Reviewer #1:**

**1- Report confidence intervals for the primary pain outcome (WBPRS) to quantify the magnitude and precision of treatment differences rather than relying primarily on nonsignificant p-values.**

Pairwise mean differences in WBPRS scores with Tukey-adjusted 95% confidence intervals are now reported in new S2 Table and Results section. All intervals included zero, and their width is acknowledged in the Limitations.

**2- Justify the presentation of means and 95% confidence intervals for ordinal outcomes, or alternatively report only summaries that are consistent with the nonparametric analyses, i.e. median, first and third quartiles.,**

The means with 95% CIs were added in the previous round in response to the reviewer's request for confidence intervals. We have retained them as supplementary descriptive statistics to facilitate comparison with previous trials, which predominantly report these scales as means, while medians (Q1-Q3) are now explicitly designated as the primary summary measures and all inferences are based on nonparametric tests. This is stated in the Statistical Analysis section and the Table 4 footnote.

**3- Provide effect size estimates for all Kruskal-Wallis analyses, not only for the FRANKL outcome, to ensure consistent reporting across outcomes.**

Effect size estimates are now reported for all Kruskal-Wallis analyses and the Statistical Analysis section has been revised accordingly.

**4- Temper the conclusion that cryotherapy represents an alternative to topical anesthetics because the study was not designed as an equivalence or noninferiority trial.,**

The conclusion has been tempered in the Abstract, Discussion, and Conclusions. Cryotherapy is now described as a promising approach requiring confirmation in non-inferiority trials, rather than as an alternative to topical anesthetics.

**5- Emphasize that the significant FRANKL finding is exploratory given the large number of secondary outcomes evaluated and the absence of multiplicity adjustment.**

The FRANKL finding is now explicitly described as exploratory due to multiple secondary outcomes without multiplicity adjustment.

**6- Clarify the exclusion criteria in the manuscript so that they are explicitly stated and consistent with the response to reviewers.**

Thank you very much for this valuable comment. In the previous revision, in line with Reviewer 3's comment, the exclusion criteria that mirrored the inclusion criteria were removed. As a result, the remaining exclusion criterion was no longer stated explicitly. It is now clearly specified in the Participants and Eligibility Criteria section: otherwise eligible children were excluded if the tooth to be treated was symptomatic (spontaneous or acute pain, swelling, or a sinus tract).

**7- Correct the missing percentages in the total row of Table 1, specifically 48% and 52%.**

It was corrected in accordace with reviewer comment.

**8- Remove the duplicated word "and" in the Statistical Analysis section.**

The duplicated “and” has been removed.

**9- Reinforce that the study may have been underpowered to detect small but clinically meaningful differences because the sample size calculation was based on a large assumed effect size.,**

-The Limitations section now links the possible lack of power, due to the large assumed effect size, to the wide confidence intervals observed for WBPRS differences, indicating that clinically meaningful differences cannot be excluded. The need for adequately powered trials is also noted in the Conclusions.
