# Supplementary material for: Pain, sensory acceptability, and treatment compliance following cryotherapy and topical anesthetics in children: A randomized clinical trial
Source: PLoS One. 2026 Nov 3;21(11):e0362074. doi: 10.1371/journal.pone.0362074 (PMC13730363; doi:10.1371/journal.pone.0362074)
Supplement: Peer review history

# Peer review: Decision Letter 0

**DOI:** 10.1371/journal.pone.0362074.r001

**Authors:** Aline Castilho

23 Sep 2026

Dear Dr. AYDIN ÇETİN,

Thank you for submitting your manuscript to PLOS One. After careful consideration, we feel that it has merit but does not fully meet PLOS One’s publication criteria as it currently stands. Therefore, we invite you to submit a revised version of the manuscript that addresses the points raised during the review process.

Please submit your revised manuscript by Nov 22 2026 11:59PM. If you will need more time than this to complete your revisions, please reply to this message or contact the journal office at plosone@plos.org. When you're ready to submit your revision, log on to https://www.editorialmanager.com/pone/ and select the 'Submissions Needing Revision' folder to locate your manuscript file.

- A letter that responds to each point raised by the academic editor and reviewer(s). You should upload this letter as a separate file labeled 'Response to Reviewers'.
- A marked-up copy of your manuscript that highlights changes made to the original version. You should upload this as a separate file labeled 'Revised Manuscript with Track Changes'.
- An unmarked version of your revised paper without tracked changes. You should upload this as a separate file labeled 'Manuscript'.

If you would like to make changes to your financial disclosure, please include your updated statement in your cover letter. Guidelines for resubmitting your figure files are available below the reviewer comments at the end of this letter.

If applicable, we recommend that you deposit your laboratory protocols in protocols.io to enhance the reproducibility of your results. Protocols.io assigns your protocol its own identifier (DOI) so that it can be cited independently in the future. For instructions see: https://journals.plos.org/plosone/s/submission-guidelines#loc-laboratory-protocols. Additionally, PLOS One offers an option for publishing peer-reviewed Lab Protocol articles, which describe protocols hosted on protocols.io. Read more information on sharing protocols at https://plos.org/protocols?utm_medium=editorial-email&utm_source=authorletters&utm_campaign=protocols.

As the corresponding author, your ORCID iD is verified in the submission system and will appear in the published article. PLOS supports the use of ORCID, and we encourage all coauthors to register for an ORCID iD and use it as well. Please encourage your coauthors to verify their ORCID iD within the submission system before final acceptance, as unverified ORCID iDs will not appear in the published article. *Only*  the individual author can complete the verification step; PLOS staff *cannot*  verify ORCID iDs on behalf of authors.

We look forward to receiving your revised manuscript.

Kind regards,

Aline Castilho

Academic Editor

PLOS One

Journal Requirements:

When submitting your revision, we need you to address these additional requirements.

1. Please ensure that your manuscript meets PLOS ONE's style requirements, including those for file naming. The PLOS ONE style templates can be found at

https://journals.plos.org/plosone/s/file?id=wjVg/PLOSOne_formatting_sample_main_body.pdf and

https://journals.plos.org/plosone/s/file?id=ba62/PLOSOne_formatting_sample_title_authors_affiliations.pdf

2. We are unable to open your Supporting Information file [S1.Data Set.sav]. Please kindly revise as necessary and re-upload.

3. Please include captions for your Supporting Information files at the end of your manuscript, and update any in-text citations to match accordingly. Please see our Supporting Information guidelines for more information: http://journals.plos.org/plosone/s/supporting-information.

4. If the reviewer comments include a recommendation to cite specific previously published works, please review and evaluate these publications to determine whether they are relevant and should be cited. There is no requirement to cite these works unless the editor has indicated otherwise.

Reviewers' comments:

Reviewer's Responses to Questions

**Comments to the Author**

1. Is the manuscript technically sound, and do the data support the conclusions?

Reviewer #1: Partly

Reviewer #2: Yes

Reviewer #3: Yes

Reviewer #4: Yes

**********

2. Has the statistical analysis been performed appropriately and rigorously?

Reviewer #1: Yes

Reviewer #2: Yes

Reviewer #3: Yes

Reviewer #4: Yes

**********

3. Have the authors made all data underlying the findings in their manuscript fully available?

The PLOS Data policy

Reviewer #1: Yes

Reviewer #2: Yes

Reviewer #3: Yes

Reviewer #4: Yes

**********

4. Is the manuscript presented in an intelligible fashion and written in standard English?

Reviewer #1: Yes

Reviewer #2: Yes

Reviewer #3: Yes

Reviewer #4: Yes

**********

Reviewer #1: Minor Statistical Issues:

1- Specify the statistical test and model assumptions used in the sample size calculation so that the reported effect size (Cohen's f = 0.50), power, and required sample size can be independently evaluated and linked to the planned primary analysis.

2- Clarify whether nonsignificant interaction terms were removed from the repeated-measures ANOVA models and whether reduced models containing only main effects were refit before conducting inference on treatment, sex, and time effects.

3- Reanalyze the pulse-rate and oxygen-saturation outcomes using reduced models if all interaction terms were nonsignificant, and report the resulting main-effect estimates and p-values from those reduced models.

4- Revise the interpretation of the pulse-rate findings because a nonsignificant group-by-time interaction does not support a conclusion that pulse-rate changes over time differed among treatment groups.

5- Report assessment of the sphericity assumption for repeated-measures ANOVA and, if violated, provide the corresponding Greenhouse-Geisser or Huynh-Feldt corrected results.

6- Report effect-size measures for repeated-measures ANOVA results, such as partial eta-squared (ηp²), to facilitate interpretation of both significant and nonsignificant findings.

7- Provide confidence intervals for treatment-group comparisons and treatment effects rather than relying primarily on p-values and descriptive statistics.

8- Avoid interpreting nonsignificant superiority tests as evidence that treatments are equivalent, comparable, or noninferior unless a formal equivalence or noninferiority framework was prespecified and analyzed.

9- Clarify whether post hoc comparisons were performed following the significant overall time effect observed for pulse rate and identify which measurement periods differed statistically.

10- Discuss the potential impact of multiple secondary outcomes and repeated hypothesis testing on the overall type I error rate.

11- Discuss the potential ceiling effect introduced by restricting eligibility to children with positive baseline FRANKL scores (3-4), as this may have limited the ability to detect clinically meaningful differences in treatment compliance.

12- Clarify whether the repeated-measures ANOVA results presented in the manuscript arise from the full factorial model or from a reduced model after evaluation of interaction terms.

13- Acknowledge that the sample size calculation was based on a large assumed effect size (Cohen's f = 0.50), which may have limited power to detect smaller but potentially clinically meaningful between-group differences.

14- Report the percentage corresponding to each sex count in Table 2, in addition to the raw counts.

15- Replace the minimum-maximum range in Table 5 with the interquartile range (IQR), as the IQR is a more appropriate measure of dispersion for ordinal variables summarized by the median.

Reviewer #2: The manuscript from Brand Aydin Çetin et al., describes a clinical study comparing the application of different agents prior to anesthetic injection to attenuate pain, anxiety and enhance comfort.

The study is well design and applied adequate methodologies to achieve the proposed objectives. The data are present in a clear and logical manner. Moreover, the introduction and discussion are well-written, presenting information that supports the investigation and the results obtained.

The data are present in a clear and logical manner. I do not have many comments regarding the pharmacology of local anesthetics. Perhaps it could be added to the introduction that local anesthetic formulations have an acidic pH, which can cause discomfort during injection.

Furthermore, I find it interesting that the results of cryotherapy are similar to those of other groups, which could make the procedure simpler and less expansive.

Finally, I suggest replacing Table 1 with a graph. I believe this would improve the presentation of the results.

Reviewer #3: Dear Authors,

Thank you for submitting the manuscript. The study in question aims to compare the effects of cryotherapy, anesthetic spray, and gel on pain, anxiety, physiological responses, taste acceptability, and treatment compliance in children aged 7-10 years undergoing their first dental treatment. After reviewing the manuscript, here are several points I consider relevant for revision and improvement:

o Table 1 presents redundant information (such as "10% lidocaine hydrochloride spray", "Locanest®", etc.) that is already clearly and fluently detailed in the Methods text (lines 144–151). Removing the table or integrating its content into the narrative text will streamline the manuscript and avoid unnecessary repetition.

o Established literature in Pediatric Dentistry and Dental Anesthesiology indicates that topical anesthetics (such as benzocaine and lidocaine) require 1 to 2 minutes of mucosal contact to achieve adequate tissue penetration and clinical efficacy (2 to 3 mm). Applying them for only 20 seconds may have resulted in sub-optimal superficial anesthesia, potentially explaining the lack of significant differences between the pharmacological groups and cooled water. The authors should justify and reference this methodological choice.

o The manuscript displays a common misconception by directly mirroring inclusion criteria into the exclusion criteria (e.g., stating age range in inclusion and the opposite in exclusion). Exclusion criteria should specify conditions in participants who meet the inclusion criteria but possess an additional factor that precludes participation (e.g., presence of acute pain, recent analgesic use, local anatomical alterations). A revision of this section is recommended.

o In the PDF version of the manuscript (pages 12–13, lines 224–233), there is a formatting glitch that compromises the clarity of parts of Table 3, making several headers and interactions (Group x Gender x Measurement) illegible. The table must be replaced with a clear, editable, high-resolution version.

o Although these terms are sometimes used interchangeably in clinical practice, consistent terminology is required in scientific writing. Since measurements were obtained using a pulse oximeter (line 173), the term "pulse rate" should be used consistently throughout the manuscript.

o Injection pain perception is directly influenced by needle gauge, length, and the volume/rate of anesthetic solution administration. Although the agent was specified (4% articaine with epinephrine), the manuscript omits the volume administered and needle specifications (e.g., 27G or 30G; short or extra-short). Providing these details is essential for study reproducibility. It would be better if the authors included the epinephrine concentration (such as 1:100,000 or 1:200,000), as it is more commonly reported.

o A patient's baseline symptomatic status (e.g., asymptomatic tooth vs. irreversible pulpitis or active pain) significantly alters pain threshold and pre-procedural anxiety. The manuscript merely states "indication for treatment of primary teeth" (line 124). It is critical to specify the clinical diagnoses included and discuss their potential confounding effect on pain perception and anxiety outcomes.

o The study employed a 4-arm parallel group design (25 different children per group). In pediatric pain and anxiety research, split-mouth or cross-over designs are often preferred as participants serve as their own controls, thereby minimizing inter-individual variability. Inherent individual differences in pain thresholds among participants may have masked significant between-group differences. This limitation should be explicitly addressed in the Limitations section (line 413).

Reviewer #4: Interesting subject matter. The study design I feel is well done, especially considering the age of the population that was assessed. Cryotherapy is definitely a novel method to use with children, but provides simple and effective methodology.

**********

what does this mean?). If published, this will include your full peer review and any attached files.

If you choose “no”, your identity will remain anonymous but your review may still be made public.

**Do you want your identity to be public for this peer review?**  For information about this choice, including consent withdrawal, please see our Privacy Policy

Reviewer #1: No

Reviewer #2: **Yes:**  Luiz Eduardo Nunes Ferreira

Reviewer #3: No

Reviewer #4: No

**********

[NOTE: If reviewer comments were submitted as an attachment file, they will be attached to this email and accessible via the submission site. Please log into your account, locate the manuscript record, and check for the action link "View Attachments". If this link does not appear, there are no attachment files.]

To ensure your figures meet our technical requirements, please review our figure guidelines: https://journals.plos.org/plosone/s/figures

You may also use PLOS’s free figure tool, NAAS, to help you prepare publication quality figures: https://journals.plos.org/plosone/s/figures#loc-tools-for-figure-preparation.

NAAS will assess whether your figures meet our technical requirements by comparing each figure against our figure specifications.

# Author comment: Author response to Decision Letter 1

**DOI:** 10.1371/journal.pone.0362074.r002

25 Sep 2026

Reviewer #1:

1- Specify the statistical test and model assumptions used in the sample size calculation so that the reported effect size (Cohen's f = 0.50), power, and required sample size can be independently evaluated and linked to the planned primary analysis.

-The sample size calculation has been described in more detail (lines 117-120). Although the primary outcome was analysed with the non-parametric Kruskal-Wallis test, the one-way ANOVA framework was used for the calculation, and the enrolled sample (n = 100) exceeded the calculated requirement by 24 participants, providing a margin for the lower efficiency of the non-parametric test. The limitation related to the assumed large effect size has been addressed in the Limitations section (lines 439-442).

• 2- Clarify whether nonsignificant interaction terms were removed from the repeated-measures ANOVA models and whether reduced models containing only main effects were refit before conducting inference on treatment, sex, and time effects.

• 12- Clarify whether the repeated-measures ANOVA results presented in the manuscript arise from the full factorial model or from a reduced model after evaluation of interaction terms.

-We thank the reviewer for these important comments. In the original submission, the repeated-measures ANOVA results were derived from the full factorial model, including group, sex, and measurement period and all their interactions. As none of the sex-related effects or interactions were significant, and sex was not a factor of interest in the study objectives, sex was removed from the model. The pulse rate and oxygen saturation data were reanalysed using a repeated-measures ANOVA with measurement period as the within-subject factor and group as the between-subject factor. The group x measurement period interaction was retained, as it directly addresses whether changes over time differed among the interventions. The revised results are presented in the Results section, lines 220-243 and Tables 2 and 3.

3- Reanalyze the pulse-rate and oxygen-saturation outcomes using reduced models if all interaction terms were nonsignificant, and report the resulting main-effect estimates and p-values from those reduced models.

-The group x time interaction was retained, as it constitutes the primary test of whether changes over time differed among the interventions; moreover, in repeated-measures ANOVA, the between-subject test of group is based on each participant's mean across time points and is therefore unaffected by this term. The main-effect estimates, effect sizes, and p-values from the revised model are reported in the Results section, lines 220-243 and Tables 2 and 3.

4- Revise the interpretation of the pulse-rate findings because a nonsignificant group-by-time interaction does not support a conclusion that pulse-rate changes over time differed among treatment groups.

-We agree that the nonsignificant group x time interaction does not support differential changes in pulse rate among the groups. The statement has been removed from the Abstract and Results, and the corresponding paragraph in the Discussion has been revised to state that pulse rate increased over time in all groups without significant differences among the interventions (lines 337-343).

5- Report assessment of the sphericity assumption for repeated-measures ANOVA and, if violated, provide the corresponding Greenhouse-Geisser or Huynh-Feldt corrected results.

-Mauchly's test indicated a violation of sphericity for both pulse rate (χ²(2) = 8.10, p = 0.017) and oxygen saturation (χ²(2) = 12.90, p = 0.002). Accordingly, Greenhouse-Geisser corrected results are now reported in the Results section (lines 223-224, 237-238).

6- Report effect-size measures for repeated-measures ANOVA results, such as partial eta-squared (ηp²), to facilitate interpretation of both significant and nonsignificant findings.

-As suggested, partial eta-squared (ηp²) values are now reported for all main effects and interactions of the repeated-measures ANOVA (lines 223-224, 237-238).

7- Provide confidence intervals for treatment-group comparisons and treatment effects rather than relying primarily on p-values and descriptive statistics.

-As recommended, 95% confidence intervals are now reported for between-group differences in pulse rate and oxygen saturation (S1 Table) and for group means of all ordinal outcomes (Table 4).

8- Avoid interpreting nonsignificant superiority tests as evidence that treatments are equivalent, comparable, or noninferior unless a formal equivalence or noninferiority framework was prespecified and analyzed.

-As the study was not designed as an equivalence or non-inferiority trial, expressions implying equivalence have been revised throughout the Abstract, Discussion, and Conclusions to state that the outcomes did not differ significantly among the groups. In addition, this has been acknowledged in the Limitations section.

9- Clarify whether post hoc comparisons were performed following the significant overall time effect observed for pulse rate and identify which measurement periods differed statistically.

-Bonferroni-adjusted pairwise comparisons showed that pulse rate increased significantly only from the pre-operative to the post-operative period (p = 0.012). As the time effect was not significant for oxygen saturation, post hoc comparisons were not indicated for this outcome.

10- Discuss the potential impact of multiple secondary outcomes and repeated hypothesis testing on the overall type I error rate.

-We agree that testing multiple secondary outcomes without adjustment increases the risk of type I error. Secondary outcomes are now described as exploratory in the Methods, and this limitation has been acknowledged in the Limitations section (lines 444-446).

11- Discuss the potential ceiling effect introduced by restricting eligibility to children with positive baseline FRANKL scores (3-4), as this may have limited the ability to detect clinically meaningful differences in treatment compliance.

-We agree that including only children with FRANKL scores of 3-4 may have produced a ceiling effect and limited the detection of differences in compliance, and that the findings may not apply to children with negative baseline behavior. This has been acknowledged in the Limitations section (lines 457-460).

13- Acknowledge that the sample size calculation was based on a large assumed effect size (Cohen's f = 0.50), which may have limited power to detect smaller but potentially clinically meaningful between-group differences.

-We acknowledge that the large assumed effect size (Cohen's f = 0.50) may have limited the power to detect smaller but clinically meaningful differences, particularly in pain perception, although the enrolled sample (n = 100) exceeded the calculated requirement (n = 76) with no loss to follow-up. This has been added to the Limitations section (lines 437-441).

14- Report the percentage corresponding to each sex count in Table 2, in addition to the raw counts.

-As suggested, the percentages corresponding to each sex count have been added to Table 1 (formerly Table 2).

15- Replace the minimum-maximum range in Table 5 with the interquartile range (IQR), as the IQR is a more appropriate measure of dispersion for ordinal variables summarized by the median.

-We thank the reviewer for this suggestion. The minimum-maximum ranges in the former Table 5 (now Table 4) have been replaced with interquartile ranges, and the table has been regenerated directly from the dataset.

Reviewer #2:

1) Perhaps it could be added to the introduction that local anesthetic formulations have an acidic pH, which can cause discomfort during injection.

-Thank you for this recommendation. A sentence on the acidic pH of local anesthetic solutions and its contribution to injection discomfort has been added to the Introduction (lines 67-70) as reference [9].

2) Furthermore, I find it interesting that the results of cryotherapy are similar to those of other groups, which could make the procedure simpler and less expansive.

-We thank the reviewer for highlighting this clinically relevant point. A sentence emphasizing the practical advantages of cryotherapy, including its low cost, ready availability, and absence of pharmacological side effects, has been added to the Discussion (lines 381,384). This aspect is also reflected in the Conclusions section.

3) I suggest replacing Table 1 with a graph. I believe this would improve the presentation of the results.

-Thank you for this suggestion. As Table 1 contained only the manufacturer and composition information of the materials, which duplicated the Methods section, it has been removed in line with Reviewer #3's comment.

Reviewer #3:

o Table 1 presents redundant information (such as "10% lidocaine hydrochloride spray", "Locanest®", etc.) that is already clearly and fluently detailed in the Methods text (lines 144–151). Removing the table or integrating its content into the narrative text will streamline the manuscript and avoid unnecessary repetition.

-As suggested, Table 1 has been removed, since its content is now described in the Methods section. The remaining tables have been renumbered accordingly.

o Established literature in Pediatric Dentistry and Dental Anesthesiology indicates that topical anesthetics (such as benzocaine and lidocaine) require 1 to 2 minutes of mucosal contact to achieve adequate tissue penetration and clinical efficacy (2 to 3 mm). Applying them for only 20 seconds may have resulted in sub-optimal superficial anesthesia, potentially explaining the lack of significant differences between the pharmacological groups and cooled water. The authors should justify and reference this methodological choice.

-We agree that the 20-second application time is shorter than the recommended 1-2 minutes and may have resulted in suboptimal mucosal anesthesia. This duration was kept identical across all groups for standardization, and injection was performed only after the child confirmed perceived numbness. This has been acknowledged in the Limitations section (lines 452-454).

o The manuscript displays a common misconception by directly mirroring inclusion criteria into the exclusion criteria (e.g., stating age range in inclusion and the opposite in exclusion). Exclusion criteria should specify conditions in participants who meet the inclusion criteria but possess an additional factor that precludes participation (e.g., presence of acute pain, recent analgesic use, local anatomical alterations). A revision of this section is recommended.

-The eligibility criteria have been revised so that the exclusion criteria no longer mirror the inclusion criteria and instead specify conditions that precluded participation among otherwise eligible children (lines 123-131).

o In the PDF version of the manuscript (pages 12–13, lines 224–233), there is a formatting glitch that compromises the clarity of parts of Table 3, making several headers and interactions (Group x Gender x Measurement) illegible. The table must be replaced with a clear, editable, high-resolution version.

-We thank the reviewer for pointing out this issue. Following the reanalysis requested by Reviewer #1, the pulse rate and oxygen saturation tables (now Tables 2 and 3) have been regenerated from the dataset as simplified, editable tables, with the statistical results reported in the text.

o Although these terms are sometimes used interchangeably in clinical practice, consistent terminology is required in scientific writing. Since measurements were obtained using a pulse oximeter (line 173), the term "pulse rate" should be used consistently throughout the manuscript.

-As measurements were obtained using a pulse oximeter, the term "pulse rate" is now used consistently throughout the manuscript.

o Injection pain perception is directly influenced by needle gauge, length, and the volume/rate of anesthetic solution administration. Although the agent was specified (4% articaine with epinephrine), the manuscript omits the volume administered and needle specifications (e.g., 27G or 30G; short or extra-short). Providing these details is essential for study reproducibility. It would be better if the authors included the epinephrine concentration (such as 1:100,000 or 1:200,000), as it is more commonly reported.

-The epinephrine concentration, the syringe and needle specifications, and the volume of anesthetic solution administered (approximately 1.5 mL) have been added to the Methods section, (lines 157-160 ).

o A patient's baseline symptomatic status (e.g., asymptomatic tooth vs. irreversible pulpitis or active pain) significantly alters pain threshold and pre-procedural anxiety. The manuscript merely states "indication for treatment of primary teeth" (line 124). It is critical to specify the clinical diagnoses included and discuss their potential confounding effect on pain perception and anxiety outcomes.

- All included teeth were clinically asymptomatic, and the indicated treatments (restorative treatment, pulpotomy, or extraction of primary maxillary molars) have been specified in the Methods (lines 129-131). The potential confounding effect of baseline symptomatic status has been discussed (lines 305-312); as pain and compliance were assessed during injection, before any treatment was initiated, this effect was minimized.

o The study employed a 4-arm parallel group design (25 different children per group). In pediatric pain and anxiety research, split-mouth or cross-over designs are often preferred as participants serve as their own controls, thereby minimizing inter-individual variability. Inherent individual differences in pain thresholds among participants may have masked significant between-group differences. This limitation should be explicitly addressed in the Limitations section (line 413).

- We agree that split-mouth and crossover designs reduce inter-individual variability; however, they were not feasible here, as a second intervention would have introduced a carry-over effect in children without previous dental experience and compromised participant blinding. We acknowledge that inter-individual differences in pain thresholds may have masked between-group differences, and this has been added to the Limitations section (lines 446-448).

Reviewer #4:

Interesting subject matter. The study design I feel is well done, especially considering the age of the population that was assessed. Cryotherapy is definitely a novel method to use with children, but provides simple and effective methodology.

-We sincerely thank the reviewer for the positive evaluation of our study and for recognizing the novelty and practicality of cryotherapy in pediatric patients.

Attachment Submitted filename: Response To Reviewers.docx

# Peer review: Decision Letter 1

**DOI:** 10.1371/journal.pone.0362074.r003

**Authors:** Aline Castilho

14 Oct 2026

Dear Dr. AYDIN ÇETİN,

Thank you for submitting your manuscript to PLOS One. After careful consideration, we feel that it has merit but does not fully meet PLOS One’s publication criteria as it currently stands. Therefore, we invite you to submit a revised version of the manuscript that addresses the points raised during the review process.

Please submit your revised manuscript by Nov 28 2026 11:59PM. If you will need more time than this to complete your revisions, please reply to this message or contact the journal office at plosone@plos.org. When you're ready to submit your revision, log on to https://www.editorialmanager.com/pone/ and select the 'Submissions Needing Revision' folder to locate your manuscript file.

- A letter that responds to each point raised by the academic editor and reviewer(s). You should upload this letter as a separate file labeled 'Response to Reviewers'.
- A marked-up copy of your manuscript that highlights changes made to the original version. You should upload this as a separate file labeled 'Revised Manuscript with Track Changes'.
- An unmarked version of your revised paper without tracked changes. You should upload this as a separate file labeled 'Manuscript'.

If you would like to make changes to your financial disclosure, please include your updated statement in your cover letter. Guidelines for resubmitting your figure files are available below the reviewer comments at the end of this letter.

If applicable, we recommend that you deposit your laboratory protocols in protocols.io to enhance the reproducibility of your results. Protocols.io assigns your protocol its own identifier (DOI) so that it can be cited independently in the future. For instructions see: https://journals.plos.org/plosone/s/submission-guidelines#loc-laboratory-protocols. Additionally, PLOS One offers an option for publishing peer-reviewed Lab Protocol articles, which describe protocols hosted on protocols.io. Read more information on sharing protocols at https://plos.org/protocols?utm_medium=editorial-email&utm_source=authorletters&utm_campaign=protocols.

As the corresponding author, your ORCID iD is verified in the submission system and will appear in the published article. PLOS supports the use of ORCID, and we encourage all coauthors to register for an ORCID iD and use it as well. Please encourage your coauthors to verify their ORCID iD within the submission system before final acceptance, as unverified ORCID iDs will not appear in the published article. *Only*  the individual author can complete the verification step; PLOS staff *cannot*  verify ORCID iDs on behalf of authors.

We look forward to receiving your revised manuscript.

Kind regards,

Aline Castilho

Academic Editor

PLOS One

Journal Requirements:

If the reviewer comments include a recommendation to cite specific previously published works, please review and evaluate these publications to determine whether they are relevant and should be cited. There is no requirement to cite these works unless the editor has indicated otherwise.

Please review your reference list to ensure that it is complete and correct. If you have cited papers that have been retracted, please include the rationale for doing so in the manuscript text, or remove these references and replace them with relevant current references. Any changes to the reference list should be mentioned in the rebuttal letter that accompanies your revised manuscript. If you need to cite a retracted article, indicate the article’s retracted status in the References list and also include a citation and full reference for the retraction notice.

[Note: HTML markup is below. Please do not edit.]

Reviewers' comments:

Reviewer's Responses to Questions

**Comments to the Author**

Reviewer #1: (No Response)

**********

2. Is the manuscript technically sound, and do the data support the conclusions?

Reviewer #1: Yes

**********

3. Has the statistical analysis been performed appropriately and rigorously?

Reviewer #1: Yes

**********

4. Have the authors made all data underlying the findings in their manuscript fully available?

The PLOS Data policy

Reviewer #1: Yes

**********

5. Is the manuscript presented in an intelligible fashion and written in standard English?

Reviewer #1: Yes

**********

Reviewer #1: Minor Statistical Issues:

1- Report confidence intervals for the primary pain outcome (WBPRS) to quantify the magnitude and precision of treatment differences rather than relying primarily on nonsignificant p-values.

2- Justify the presentation of means and 95% confidence intervals for ordinal outcomes, or alternatively report only summaries that are consistent with the nonparametric analyses, i.e. median, first and third quartiles.

3- Provide effect size estimates for all Kruskal-Wallis analyses, not only for the FRANKL outcome, to ensure consistent reporting across outcomes.

4- Temper the conclusion that cryotherapy represents an alternative to topical anesthetics because the study was not designed as an equivalence or noninferiority trial.

5- Emphasize that the significant FRANKL finding is exploratory given the large number of secondary outcomes evaluated and the absence of multiplicity adjustment.

6- Clarify the exclusion criteria in the manuscript so that they are explicitly stated and consistent with the response to reviewers.

7- Correct the missing percentages in the total row of Table 1, specifically 48% and 52%.

8- Remove the duplicated word "and" in the Statistical Analysis section.

9- Reinforce that the study may have been underpowered to detect small but clinically meaningful differences because the sample size calculation was based on a large assumed effect size.

**********

what does this mean?). If published, this will include your full peer review and any attached files.

If you choose “no”, your identity will remain anonymous but your review may still be made public.

**Do you want your identity to be public for this peer review?**  For information about this choice, including consent withdrawal, please see our Privacy Policy

Reviewer #1: No

**********

[NOTE: If reviewer comments were submitted as an attachment file, they will be attached to this email and accessible via the submission site. Please log into your account, locate the manuscript record, and check for the action link "View Attachments". If this link does not appear, there are no attachment files.]

To ensure your figures meet our technical requirements, please review our figure guidelines: https://journals.plos.org/plosone/s/figures

You may also use PLOS’s free figure tool, NAAS, to help you prepare publication quality figures: https://journals.plos.org/plosone/s/figures#loc-tools-for-figure-preparation.

NAAS will assess whether your figures meet our technical requirements by comparing each figure against our figure specifications.

# Author comment: Author response to Decision Letter 2

**DOI:** 10.1371/journal.pone.0362074.r004

15 Oct 2026

14th October, 2026

Dear Editor Dr. Aline Castilho and Reviewer,

We sincerely thank the Academic Editor and the reviewer for their time and for their careful and constructive evaluation of our manuscript. We have addressed each comment point by point below. The reviewer’s comments are shown in bold, followed by our responses.

Journal requirements: The reference list was checked; no retracted articles are cited.

Reviewer #1:

1- Report confidence intervals for the primary pain outcome (WBPRS) to quantify the magnitude and precision of treatment differences rather than relying primarily on nonsignificant p-values.

Pairwise mean differences in WBPRS scores with Tukey-adjusted 95% confidence intervals are now reported in new S2 Table and Results section. All intervals included zero, and their width is acknowledged in the Limitations.

2- Justify the presentation of means and 95% confidence intervals for ordinal outcomes, or alternatively report only summaries that are consistent with the nonparametric analyses, i.e. median, first and third quartiles.,

The means with 95% CIs were added in the previous round in response to the reviewer's request for confidence intervals. We have retained them as supplementary descriptive statistics to facilitate comparison with previous trials, which predominantly report these scales as means, while medians (Q1-Q3) are now explicitly designated as the primary summary measures and all inferences are based on nonparametric tests. This is stated in the Statistical Analysis section and the Table 4 footnote.

3- Provide effect size estimates for all Kruskal-Wallis analyses, not only for the FRANKL outcome, to ensure consistent reporting across outcomes.

Effect size estimates are now reported for all Kruskal-Wallis analyses and the Statistical Analysis section has been revised accordingly.

4- Temper the conclusion that cryotherapy represents an alternative to topical anesthetics because the study was not designed as an equivalence or noninferiority trial.,

The conclusion has been tempered in the Abstract, Discussion, and Conclusions. Cryotherapy is now described as a promising approach requiring confirmation in non-inferiority trials, rather than as an alternative to topical anesthetics.

5- Emphasize that the significant FRANKL finding is exploratory given the large number of secondary outcomes evaluated and the absence of multiplicity adjustment.

The FRANKL finding is now explicitly described as exploratory due to multiple secondary outcomes without multiplicity adjustment.

6- Clarify the exclusion criteria in the manuscript so that they are explicitly stated and consistent with the response to reviewers.

Thank you very much for this valuable comment. In the previous revision, in line with Reviewer 3's comment, the exclusion criteria that mirrored the inclusion criteria were removed. As a result, the remaining exclusion criterion was no longer stated explicitly. It is now clearly specified in the Participants and Eligibility Criteria section: otherwise eligible children were excluded if the tooth to be treated was symptomatic (spontaneous or acute pain, swelling, or a sinus tract).

7- Correct the missing percentages in the total row of Table 1, specifically 48% and 52%.

It was corrected in accordace with reviewer comment.

8- Remove the duplicated word "and" in the Statistical Analysis section.

The duplicated “and” has been removed.

9- Reinforce that the study may have been underpowered to detect small but clinically meaningful differences because the sample size calculation was based on a large assumed effect size.,

-The Limitations section now links the possible lack of power, due to the large assumed effect size, to the wide confidence intervals observed for WBPRS differences, indicating that clinically meaningful differences cannot be excluded. The need for adequately powered trials is also noted in the Conclusions.

Attachment Submitted filename: Response to the Reviewer.docx

# Peer review: Decision Letter 2

**DOI:** 10.1371/journal.pone.0362074.r005

**Authors:** Aline Castilho

21 Oct 2026

Pain, Sensory Acceptability, and Treatment Compliance Following Cryotherapy and Topical Anesthetics in Children: A Randomized Clinical Trial

PONE-D-26-37677R2

Dear Dr. AYDIN ÇETİN,

We’re pleased to inform you that your manuscript has been judged scientifically suitable for publication and will be formally accepted for publication once it meets all outstanding technical requirements.

Within one week, you’ll receive an e-mail detailing the required amendments. When these have been addressed, you’ll receive a formal acceptance letter and your manuscript will be scheduled for publication.

An invoice will be generated when your article is formally accepted. Please note, if your institution has a publishing partnership with PLOS and your article meets the relevant criteria, all or part of your publication costs will be covered. Please make sure your user information is up-to-date by logging into Editorial Manager at Editorial Manager® and clicking the ‘Update My Information' link at the top of the page. For questions related to billing, please contact billing support.

If your institution or institutions have a press office, please notify them about your upcoming paper to help maximize its impact. If they’ll be preparing press materials, please inform our press team as soon as possible -- no later than 48 hours after receiving the formal acceptance. Your manuscript will remain under strict press embargo until 2 pm Eastern Time on the date of publication. For more information, please contact onepress@plos.org.

Kind regards,

Aline Castilho

Academic Editor

PLOS One

Additional Editor Comments (optional):

Reviewers' comments:

Reviewer's Responses to Questions

**Comments to the Author**

Reviewer #1: All comments have been addressed

**********

2. Is the manuscript technically sound, and do the data support the conclusions?

Reviewer #1: (No Response)

**********

3. Has the statistical analysis been performed appropriately and rigorously?

Reviewer #1: (No Response)

**********

4. Have the authors made all data underlying the findings in their manuscript fully available?

The PLOS Data policy

Reviewer #1: (No Response)

**********

5. Is the manuscript presented in an intelligible fashion and written in standard English?

Reviewer #1: (No Response)

**********

Reviewer #1: All comments have been adequately addressed.

**********

what does this mean?). If published, this will include your full peer review and any attached files.

If you choose “no”, your identity will remain anonymous but your review may still be made public.

**Do you want your identity to be public for this peer review?**  For information about this choice, including consent withdrawal, please see our Privacy Policy

Reviewer #1: No

**********

# Editor report: Acceptance letter

**DOI:** 10.1371/journal.pone.0362074.r006

**Authors:** Aline Castilho

PONE-D-26-37677R2

PLOS One

Dear Dr. AYDIN ÇETİN,

I'm pleased to inform you that your manuscript has been deemed suitable for publication in PLOS One. Congratulations! Your manuscript is now being handed over to our production team.

At this stage, our production department will prepare your paper for publication. This includes ensuring the following:

* All references, tables, and figures are properly cited

* All relevant supporting information is included in the manuscript submission,

* There are no issues that prevent the paper from being properly typeset

You will receive further instructions from the production team, including instructions on how to review your proof when it is ready. Please keep in mind that we are working through a large volume of accepted articles, so please give us a few days to review your paper and let you know the next and final steps.

Lastly, if your institution or institutions have a press office, please let them know about your upcoming paper now to help maximize its impact. If they'll be preparing press materials, please inform our press team within the next 48 hours. Your manuscript will remain under strict press embargo until 2 pm Eastern Time on the date of publication. For more information, please contact onepress@plos.org.

You will receive an invoice from PLOS for your publication fee after your manuscript has reached the completed accept phase. If you receive an email requesting payment before acceptance or for any other service, this may be a phishing scheme. Learn how to identify phishing emails and protect your accounts at https://explore.plos.org/phishing.

If we can help with anything else, please email us at customercare@plos.org.

Thank you for submitting your work to PLOS One and supporting open access.

Kind regards,

PLOS One Editorial Office Staff

on behalf of

Dr. Aline Castilho

Academic Editor

PLOS One
